# Exercise performance in well‐trained male mice is promoted by intermittent hyperoxia via improving metabolic properties and capillary profiles

**DOI:** 10.14814/phy2.70341

**Published:** 2025-04-22

**Authors:** Junichi Suzuki

**Affiliations:** ^1^ Laboratory of Exercise Physiology, Health and Sports Sciences, Course of Sports Education, Department of Education Hokkaido University of Education Midorigaoka, Iwamizawa Hokkaido Japan

**Keywords:** antioxidant enzyme, Bayesian data analysis, fatty acid metabolism, hybrid exercise, intermittent hyperoxia

## Abstract

Although training under intermittent hyperoxia has been shown to improve exercise performance, its effect on well‐trained mice remains undetermined. Voluntary run for 7 weeks increased maximal work values by 7.4‐fold (Bayes factor, BF ≥ 30). Subsequently, mice underwent 4 weeks of treadmill training with (INT) or without (ET) intermittent hyperoxia (30% O_2_). INT training significantly increased maximal exercise capacity compared to ET (BF ≥ 30). INT group exhibited significantly higher levels of cytochrome‐c‐oxidase (COX) in soleus muscle (SOL, BF ≥ 3.0). Additionally, INT enhanced 3‐hydroxyacyl‐CoA‐dehydrogenase (HAD) levels in white gastrocnemius (Gw) and plantaris (PL) muscles compared to ET (BF ≥ 3.0). Pyruvate dehydrogenase complex (PDHc) levels were significantly higher in the INT group compared to the ET group in red gastrocnemius and left ventricle (BF ≥ 30). Capillary‐to‐fiber ratio (C/F) was significantly higher in the INT group than in the ET group in SOL and PL muscles (BF ≥ 3.0). COX, PDHc, capillary density (CD), and catalase protein values in SOL, HAD, and C/F levels in Gw and PL, as well as CD values in Gw showed a significant positive correlation with maximal work values using data from ET and INT groups (*p* < 0.05). These findings suggest that training under intermittent hyperoxia promotes endurance performance probably by improving metabolic enzyme levels and capillary profiles in well‐trained mice.

## 1 INTRODUCTION

More recently, hyperoxic training, which involves exercise in a hyperoxic environment, has gained popularity among athletes and has been extensively studied to understand its performance‐enhancing effects. Exercise under 70% oxygen at the same absolute workload as normoxia showed lower levels of ventilation, heart rate, blood lactate, and catecholamines (Byrnes et al., 1984). Cycling exercise with 50% oxygen improved maximal power output and endurance capacity in humans (Ulrich et al., 2017). These findings suggest that daily exercise training under hyperoxia or intermittent hyperoxia may be a less physically demanding procedure.

Cellular responses to hypoxia are well documented. Under hypoxic conditions, hypoxia‐inducible factor (HIF) 1α is stabilized and translocated to the nucleus, leading to the upregulation of HIF1α‐responsive genes (Maxwell et al., 1999). Chronic stabilization of HIF1α has been shown to increase glycolytic enzyme activity and decrease lipid oxidation (Kennedy et al., 2001), likely aiding survival in low‐oxygen environments. The protein levels of HIF1α are regulated by prolyl hydroxylases (PHDs), which play a crucial role in degrading HIF1α through hydroxylation (Bruick & McKnight, 2001). In response to an acute bout of endurance exercise, HIF1α is stabilized in skeletal muscle (Ameln et al., 2005). However, after long‐term endurance training, the HIF1α response to exercise is attenuated due to increased expression of negative regulators of the HIF system (Richardson et al., 2000), such as PHDs and factor inhibiting HIF (FIH) (Lindholm et al., 2014). This inhibition likely promotes oxidative metabolism, thereby facilitating endurance exercise performance (Lindholm & Rundqvist, 2016).

It has been proposed that hyperoxic exposure followed by normoxia is interpreted as a hypoxic event at the cellular level, a phenomenon known as the hyperoxic‐hypoxic paradox (Salvagno et al., 2023). Thus, repeated hyperoxic and normoxic exposure may stabilize HIF1α (Balestra et al., 2006; Cimino et al., 2012). In humans, breathing 100% oxygen for 2 h, followed by 36 h of room air, resulted in a 60% increase in serum erythropoietin levels (Balestra et al., 2006). Acute intermittent hyperoxic exposure (three cycles of 21% O_2_ for 10 min and 30% O_2_ for 15 min), followed by 3 h of rest in room air, upregulated HIF1α target genes (phosphofructokinase [PFK] and vascular endothelial growth factor [VEGF]), as well as genes known to promote muscle metabolic properties (mitochondrial transcription factor A, peroxisome proliferator‐activated receptors‐α and ‐γ) (Suzuki, 2024).

Recently, studies have shown that reactive oxygen species (ROS) play a role in stabilizing and activating HIF1α, which in turn regulates gene expression (Schumacker, 2011). Both hypoxic and hyperoxic conditions have been found to increase ROS production (Clanton, 2007). The formation of ROS in mitochondria has been reported to rise linearly with increasing levels of inhaled oxygen (Turrens, 2003). Exercise has been shown to enhance ROS production, particularly in active tissues (Trewin et al., 2018). To date, no negative effects of short‐term inhalation of hyperoxic air during exercise or post‐exercise recovery in humans. After all‐out interval exercise, serum oxidative stress markers were markedly increased comparably in both the 21% and 60% O_2_ conditions (Kon et al., 2021). Supplemental oxygen (99.5% O_2_) provided during the recovery periods of interval exercise had no effect on post‐exercise ROS levels or inflammatory responses (White et al., 2013). Increased ROS levels during hyperoxia are likely to diminish upon returning to normoxia (Balestra et al., 2006). Therefore, the repeated normoxia/hyperoxia cycle in the present exercise training with short‐duration intermittent hyperoxia (hereinafter referred to as INT training) may stimulate additional ROS production. ROS has been shown to facilitate exercise‐induced muscle adaptation (Allen & Tresini, 2000). However, excess ROS production may damage cellular proteins, DNA, and lipids (Powers et al., 2023). Thus, the role of the antioxidant system is not to eliminate ROS completely but to regulate them at appropriate levels (Lu et al., 2021). Muscle fibers and other cells contain endogenous enzymatic and nonenzymatic antioxidants that can eliminate ROS (Vincent et al., 2000). If these antioxidant levels are maintained after INT training, it is likely that both facilitating muscle adaptation and prevention of oxidative damage will be achieved. The oxidative stress induced by severe exercise is transient and is likely restored to normal levels within 24 h due to the stimulated endogenous antioxidant system (Lu et al., 2021). In the present study, tissue samples were obtained 48 h after post‐testing. Thus, the effects of INT training on antioxidant levels were assessed by the expression levels of representative antioxidant proteins (Powers & Lennon, 1990), including superoxide dismutase‐1 (SOD1), catalase (CAT), and glutathione peroxidase‐1/2 (GPX1/2), rather than plasma antioxidant stress markers.

A previous study was the first to demonstrate that INT training combined with endurance exercise (6 days per week, 30% O_2_) on alternating days for 4 weeks has additional benefits for improving endurance performance in mice (Suzuki, 2024). To further investigate the effects of INT training on endurance performance in athletes, additional experiments using well‐trained animals are likely to provide valuable evidence. In college male athletes, high‐intensity interval training under hyperoxia (60% O_2_) did not improve maximal oxygen uptake values (Kon et al., 2019, 2020). Thus, any ergogenic effect of hyperoxic training has yet to be observed in well‐trained athletes. In mice housed in a wheel activity device for 7 weeks starting at the age of 5 weeks, endurance performance was enhanced by approximately 6.8‐fold (Suzuki, 2021), but interval exercise performance, assessed by a 10‐s run followed by a 10‐s rest at 10–55 m min^−1^, did not improve (Suzuki, 2019a). After these trained mice underwent forced treadmill exercise for 4 weeks, no further improvements in endurance performance were observed (Suzuki, 2019a, 2019b, 2021). Thus, the maximal level of endurance performance has likely been achieved in the mice trained with a 7‐week wheel run from a young age. Potential benefits of INT training for athletes were likely highlighted by an experiment using these mice.

In the present study, metabolic enzyme levels, muscle fiber proportions, and capillary distributions were determined in calf muscles (soleus [SOL], plantaris [PL], red gastrocnemius [Gr], and white gastrocnemius [Gw]). In rats, muscle blood flow during treadmill walking (15 m min^−1^) significantly increased from resting values in the soleus (SOL, [+275%]), plantaris (PL, [+63%]), and red gastrocnemius (Gr, [+475%]), but remained unchanged in the white gastrocnemius (Gw) (Delp et al., 1989). Furthermore, after endurance training, these values significantly increased in SOL, PL, and Gr (Armstrong & Laughlin, 1984), as well as in fast‐twitch white muscle regions of calf muscles by 40%–50% (Mackie & Terjung, 1983). Thus, almost all portions of the calf muscles likely contribute to endurance training, but the extent of contribution to improving maximal performance likely varies among muscle types or regions.

High‐intensity interval exercise (6 × 30‐s all‐out cycling) followed by endurance exercise (60% VO_2_max for 60 min) markedly enhanced mRNA levels of PGC‐1α (at 3 h after exercise) compared to those observed after the respective single regimens in trained human muscle (Skovgaard et al., 2016). Thus, this hybrid training protocol may improve muscle metabolic properties and exercise performance. Furthermore, adaptive changes induced by INT with hybrid exercises would be observed in a tissue‐dependent manner, particularly in calf muscles, which have a wide variety of properties, as well as in the diaphragm and the heart.

In the present study, experiments were designed to elucidate the effects of chronic exercise training under intermittent hyperoxia on endurance capacity as well as muscle metabolic features, muscle capillary and fiber type properties, and antioxidant protein levels in well‐trained mice.

## 2 MATERIALS AND METHODS

### 2.1 Ethical approval

All procedures were approved by the Animal Care and Use Committee of Hokkaido University of Education (approved on 2024/4/3) and conducted in accordance with the “Guiding Principles for the Care and Use of Animals in the Field of Physiological Sciences” of the Physiological Society of Japan.

### 2.2 Animals

**FIGURE 1:**
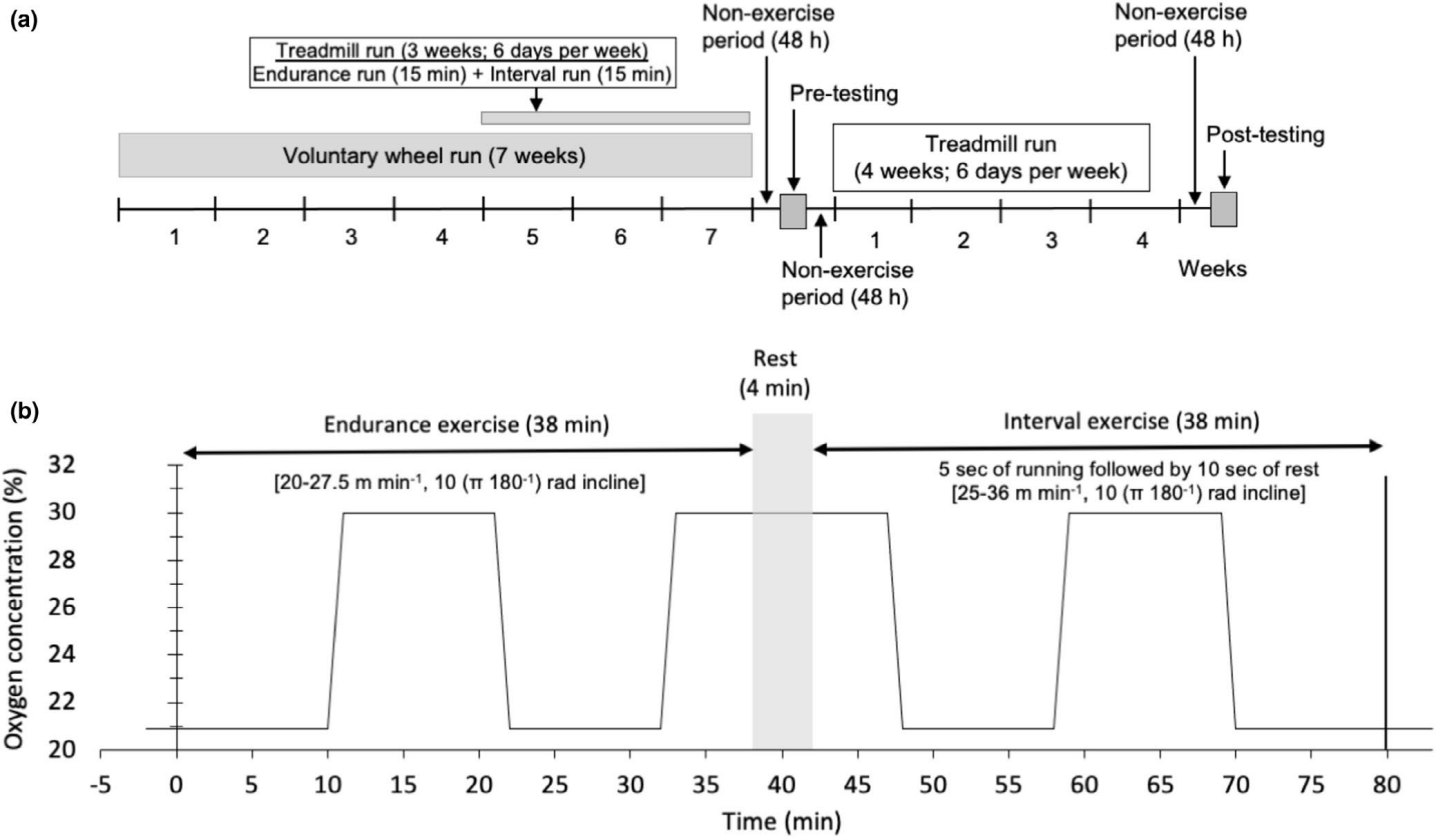
Experimental design. (a) Whole experimental protocols; (b) Daily experimental protocols.

The present experimental design was summarized in Figure 1. Thirty male MCH(ICR)/jcl mice, aged 3 weeks, were purchased from Clea Japan (Tokyo, Japan). They were housed under controlled conditions, with a temperature of 24 ± 1°C and a relative humidity of approximately 50%. Lighting was automatically controlled from 7:00 to 19:00. All mice were given commercial laboratory chow (solid CE‐2; Clea Japan) and tap water ad libitum. After the mice had been fed for 2 weeks and allowed to adapt to the new environment, they were randomly assigned to a sedentary control group (SED, *n* = 10) or training group (*n* = 20).

#### 2.2.1 Voluntary wheel run

**FIGURE 2:**
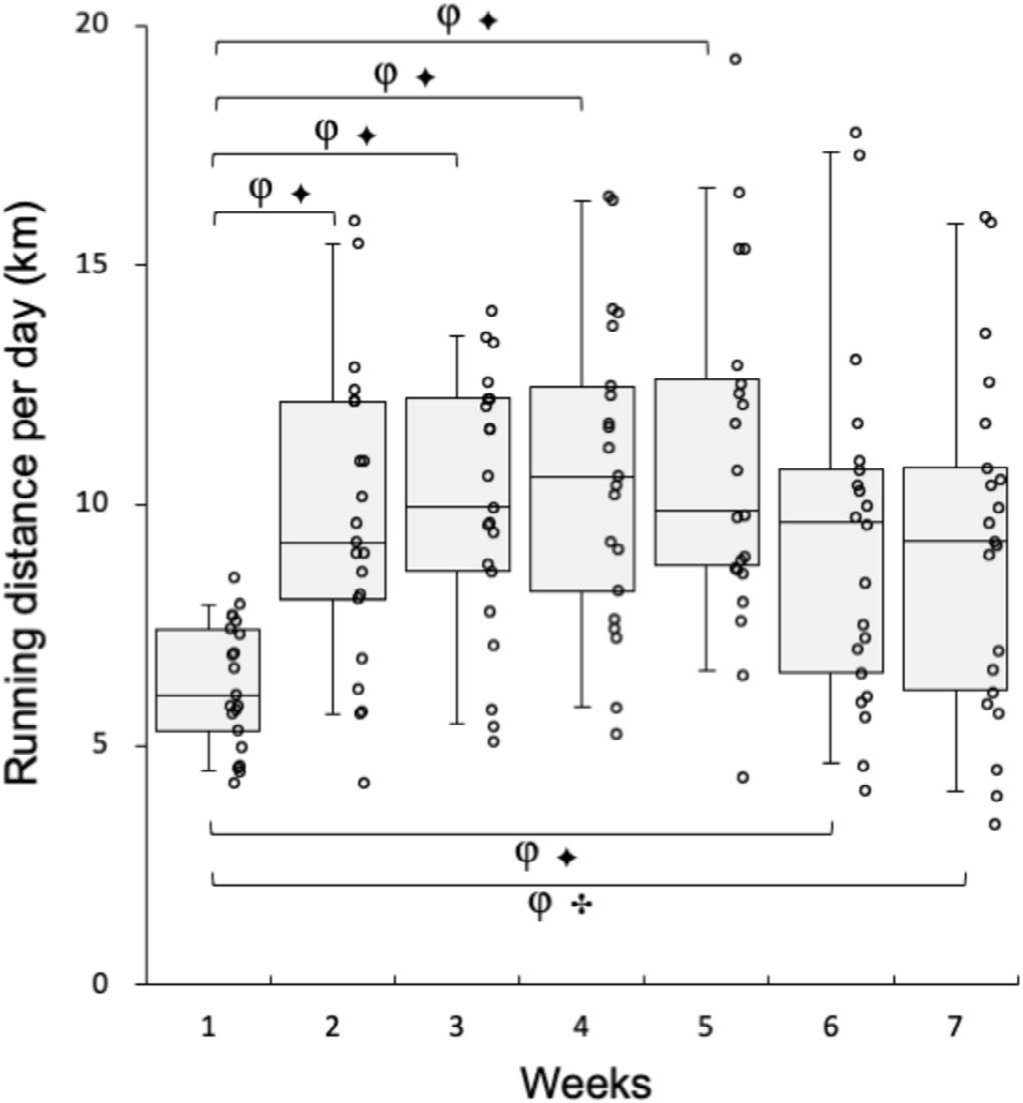
Running distance per day during voluntary wheel training. Values are expressed as box and whisker plots with 5th, 25th, 50th, 75th, and 95th percentile. Dots in the figure indicate data for each mouse. Bayes factor: ✣, ≥10; ✦, ≥30. φ, The 95% confidential interval did not contain the mean value of target group for comparison.

Mice in the training group were individually housed in a cage with a wheel activity device (13 cm in diameter) for 7 weeks (Figure 1a). Wheel activity (distance and running time) was monitored and recorded using digital bike computers (CC‐VL820; Cateye, Osaka Japan). Running distance per day during voluntary wheel training was shown in Figure 2. To familiarize mice with a treadmill device, all mice including those in the SED group were subjected to walking once a week on a controlled treadmill (Modular motor assay; Columbus Instruments, Columbus, OH, USA) for 3 min per day at 10–15 m min^−1^ with a 5 (π 180^−1^) rad incline.

To familiarize mice with running on a treadmill and an interval exercise regimen, mice in the training group were subjected to treadmill exercise using a treadmill device (KN‐73, Natsume, Tokyo, Japan) during the 5th through 7th weeks of voluntary running. The regimen consisted of 15 min of endurance running, followed by a 2‐min rest, and then an interval run (5 s of running followed by 10 s of rest) for an additional 15 min. In the 5th week, the treadmill had a 7.5 (π 180^−1^) rad incline, which increased to 10 (π 180^−1^) rad from the 6th week onward. The speed for the endurance run was set at 15 m min^−1^ during the 5th and 6th weeks and increased to 20 m min^−1^ in the 7th week. For the interval run, the speed was 20 m min^−1^ in the 5th week and 25 m min^−1^ from the 6th week onward.

#### 2.2.2 Maximal exercise capacity test (pre‐testing)

Following the voluntary training, the mice were given a 48‐h non‐exercise period before the maximal exercise capacity test. This test was conducted using a graded ramp running protocol on the controlled treadmill, as previously reported (Suzuki, 2022). Total work (J) was calculated by multiplying body weight (kg), speed (m s^−2^), time (sec), slope (%), and 9.8 (m s^−2^). Exhaustion was defined as the condition in which the mouse remained on the metal grid at the rear of the treadmill for more than 5 s (without electrical shock), despite gentle external stimulation applied to the tail with a bamboo stick (0.8 mm in diameter).

#### 2.2.3 Hybrid exercise training

**FIGURE 3:**
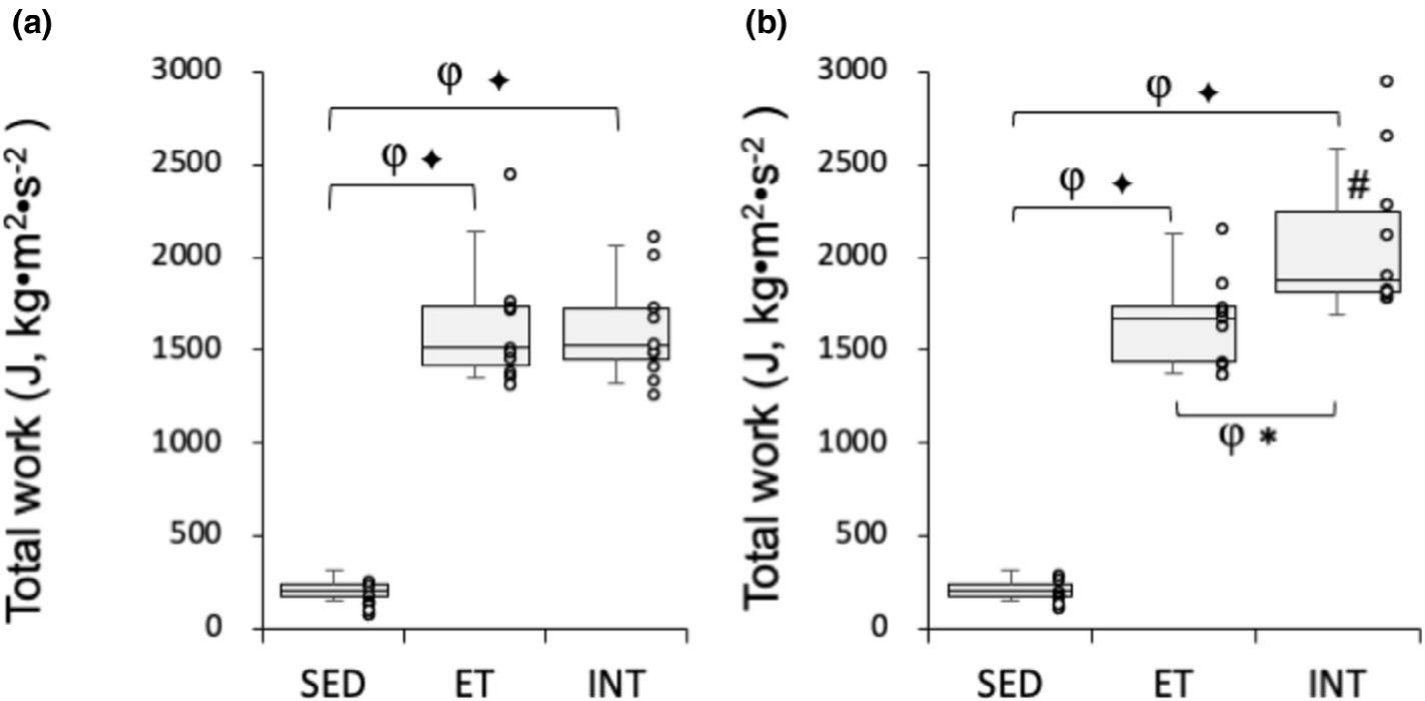
Results of endurance exercise performance test. Total work values of the endurance capacity test (a) before and (b) after 4 weeks of treadmill exercise training. Values are expressed as box and whisker plots with 5th, 25th, 50th, 75th, and 95th percentile. Dots in the figure indicate data for each mouse. #, significantly different (BF ≥ 15) from pre‐treadmill training values of each group shown in the panel (a). Bayes factor: ✻, ≥3; ✣, ≥10; ✦, ≥30. φ, The 95% confidential interval did not contain the mean value of target group for comparison.

After the test, mice in the training group underwent exercise training for 4 weeks (Figure 1b). Voluntary trained mice were divided into a normoxic exercise‐trained group (ET, *n* = 10) and an exercise‐trained under intermittent hyperoxia group (INT, *n* = 10) to match the mean and standard deviation (SD) values for total work (Mean ± SD, ET, 1629.2 ± 321.8; INT, 1615.0 ± 269.9, Figure 3a). All mice in the trained groups successfully completed the exercise training, and their results were included in this study.

The mice in the training groups underwent hybrid exercise training, as shown in Figure 1b, for 4 weeks, 6 days a week. The hybrid exercise lasted for 80 min and consisted of an endurance run for 38 min, followed by 4 min of rest on the treadmill, and interval exercise for 38 min, with a 10 (π 180^−1^) rad incline. Instead of using electrical shock, the mice were motivated to run by gently touching their tail or planta pedis with a conventional test tube brush made of soft porcine bristles if they remained on a metal grid for more than 3 s.

On the first day, the mice ran at 20 m min^−1^ for endurance exercise. The speed was gradually increased to 25 m min^−1^ and 27.5 m min^−1^ on the 7th and 10th days of the training period, respectively. For interval exercise (5 sec of running followed by 10 sec of rest), the mice ran at 25 m min^−1^ on the first day. The speed was gradually increased to 27.5 m min^−1^, 29 m min^−1^, 30.5 m min^−1^, 32 m min^−1^, 34 m min^−1^, and 36 m min^−1^ on the 2nd, 4th, 7th, 10th, 13th, and 16th days of the training period, respectively. Each exercise intervention took place between 5 am and 9 am, and the order of the interventions was randomized daily.

#### 2.2.4 Exercise with intermittent hyperoxia

The INT group exercised under intermittent hyperoxia, consisting of three cycles of 30% O_2_ for 15 min, followed by room air for 10 min, as illustrated in Figure 1b, using a treadmill (KN‐73). During the hyperoxia sessions, the treadmill runway was covered with a translucent plastic film to create a runway chamber, measuring 1.10 m in length, 0.78 m in width, and 0.3 m in height. To maintain CO_2_ concentrations below 1000 ppm, air in the chamber was circulated through a CO_2_ absorbent (Litholyme; Allied Healthcare Products, St. Louis, MO, USA).

When the O_2_ concentration was increased from 21% to 30%, 100% O_2_ was introduced into an air‐mixture box (length 0.32 m, width 0.17 m, and height 0.10 m) installed within the runway chamber. Inside the air‐mixture box, 100% O_2_ was mixed with circulated air (180 L min^−1^, using three closed air circulating pumps, VP6035S, Techno Takatsuki, Osaka, Japan) for CO_2_ absorption, as previously described (Suzuki, 2024). The mixed air was then introduced into the runway chamber via a commercial circulator, ensuring that 100% O_2_ was not directly introduced into the chamber.

The O_2_ concentrations in both the air‐mixture box and the runway chamber were monitored using two sets of oxygen analyzers (G‐1690 and GOX‐100; Greisinger, Germany). When the O_2_ concentration was reduced from 30% to 21%, room air was introduced into the chamber by opening the runway chamber while simultaneously removing air from the chamber using a combined vacuum cleaner (MCU21, Panasonic, Osaka, Japan). Additionally, room air in the laboratory was continuously ventilated through a draft chamber (DF17C, Dalton, Tokyo, Japan).

#### 2.2.5 Post‐testing and sampling

The maximal endurance capacity test was performed 48 h after the last run, as described above. Forty‐eight hours after the performance test, the mice were anesthetized with 3% sevoflurane (193‐17791; Fujifilm‐Wako, Osaka, Japan) inhalation using an anesthetizer (MKA100W, Muromachi Kikai, Tokyo, Japan), and the adequacy of anesthesia was validated using a toe pinch response. The soleus (SOL), plantaris (PL), and gastrocnemius muscles were excised, and the deep red region (Gr) of the gastrocnemius was separated from the superficial white region (Gw). The diaphragm (DIA) was also excised. All samples were frozen in liquid nitrogen for biochemical analyses. The mice were killed by excision of the heart. After excision, the whole heart and left ventricle (LV) were weighed. All tissue samples were stored at −80°C until further analyses.

### 2.3 Sample preparation for biochemical analyses

A cytoplasmic or nuclear fraction of protein was obtained separately using precisely the same protocols as previously reported by the author (Suzuki, 2022). The efficacy of the separation was confirmed previously (Suzuki, 2022) by Western blot using anti‐GAPDH antibody (a cytoplasmic marker, sc‐166,574; Santa Cruz Biotechnology, Dallas, TE, USA) and anti‐Lamin A/C antibody (a nuclear marker, sc‐376248; Santa Cruz). Images of previously published data are provided by public Figures S1–S10.

Frozen tissue powder was obtained using a frozen sample crusher (SK mill; Tokken, Chiba, Japan) and homogenized with ice‐cold medium (10 mM HEPES buffer, pH 7.4; 1% NP‐40 [Fujifilm‐Wako]; 11.5% [w/v] sucrose; and 5% [v/v] protease inhibitor cocktail [P2714; Sigma‐Aldrich, St. Louis, MD, USA]) in an ultrasonic bath (43 kHz, 50 W) at 4°C for 5 min. It was then gently rotated at 4°C for 10 min. After centrifugation at 18,000*g* and at 4°C for 10 min, the supernatant, cytoplasmic fraction, was collected and stored at −80°C. The pellet was resuspended with ice‐cold buffer described above without NP‐40 and rotated at 4°C for 10 min. After centrifugation as described above, the supernatant was drained, and the pellet was resuspended with ice‐cold buffer (10 mM HEPES buffer, pH 7.4; 1.5 mM MgCl_2_, 420 mM NaCl, 25% glycerol, and 5% [v/v] protease inhibitor cocktail). It was then rotated at 4°C for 10 min. After centrifugation at 18,000*g* and at 4°C for 10 min, the supernatant, nuclear extraction, was used for Western blot analysis. Total protein concentrations were measured using the Bradford assay (0.01% [w/v] CBB G‐250 [B3193, Tokyo Chemical Industry, Tokyo, Japan], 5% [v/v] ethanol [054‐07225, Fujifilm‐Wako, Osaka, Japan], 8.5% [v/v] phosphoric acid [164‐02176, Fujifilm‐Wako]) with bovine serum albumin (21011, iNtRON Biotechnology, Korea) as a standard.

### 2.4 Western blot analyses

A sample containing 30 μg of nuclear protein or 50 μg of cytoplasmic protein was heated at 99°C for 5 min with a Laemmli sample buffer (161‐0747, Bio‐Rad Laboratories, Hercules, CA, USA.). Then the sample was separated on 12% polyacrylamide gels (TGX StainFree FastCast gel, 1610185, Bio‐Rad) using SDS/PAGE. The gels were exposed to ultra‐violet (UV) light for 1 min, and total protein patterns were visualized using the ChemiDoc MP Imager (Bio‐Rad). The stain‐free gel contains a trihalo compound that reacts with proteins during separation, making them detectable with UV exposure (Gilda & Gomes, 2013). The gels were then electrophoretically transferred to a polyvinylidene fluoride membrane. The blot was blocked with 5% nonfat dry milk (190‐12865; Fujifilm‐Wako) in 0.1 M phosphate‐buffered saline (PBS) with 0.05% Tween20 for 1 h. Next, the blot was exposed to a specific primary antibody against NT‐PGC1α (1:1000, sc‐518,025, Santa Cruz Biotechnology, Dallas, TE, USA) for the nuclear sample, CAT (1:1500, sc‐271803, Santa Cruz), SOD1 (1:2000, sc‐101523, Santa Cruz), or GPx1/2 (1:750, sc‐133160, Santa Cruz) for the cytoplasmic sample diluted in blocking buffer for 1 h. According to the molecular weight of the detected specific bands (Figure S1, #7‐9), GPX1 protein was observed in the current procedures. After incubating the blot with a HRP‐labeled mouse IgGκ light chain binding protein (1:6000, sc‐516102, Santa Cruz), it was reacted with Clarity Western ECL substrate (1705060, Bio‐Rad), Clarity Max Western ECL substrate (1705062, Bio‐Rad), or a mixture of both. The target proteins were detected with the ChemiDoc MP (Bio‐Rad). The densities of the specific bands were quantified using Image Lab software (Bio‐Rad) and normalized to the densities of all protein bands in each lane on the membrane (Suzuki, 2021). Complete images for all protein bands and specific bands of the target protein detected after immunostaining are shown in Figure S1. This normalizing procedure was confirmed to be superior to using β‐actin as a loading control (Gilda & Gomes, 2013). Subsequently, the normalized densities of the bands were further normalized to the same sample that was run on every gel and transferred to every membrane, as reported by the author (Suzuki, 2021).

### 2.5 Biochemical analyses of enzyme activity

The activity of 3‐hydroxyacyl‐CoA‐dehydrogenase (HAD) was assayed using the method described by Bass et al. (1969). Assays for cytochrome c oxidase (COX) activities were prepared according to the method of Sherratt et al. (1988). The activity of citrate synthase (CS) was assayed following the methods of Srere (1969). Pyruvate dehydrogenase complex (PDHc) activity was assayed according to the method of Ke et al. (2014). The activity of carnitine palmitoyl transferase (CPT) 2 was assayed as previously reported by Suzuki (2021). Specific lactate dehydrogenase activities, pyruvate‐to‐lactate (LDH‐PL) or lactate‐to‐pyruvate (LDH‐LP) conversions, were determined following the protocol of Howell et al. (1979) with some modifications as reported previously (Suzuki, 2022). All measurements were carried out at 25°C using a spectrophotometer (U‐2001; Hitachi Co., Tokyo, Japan), and enzyme activities were reported as micromoles per hour per milligram of protein.

### 2.6 Histological analyses

**FIGURE 4:**
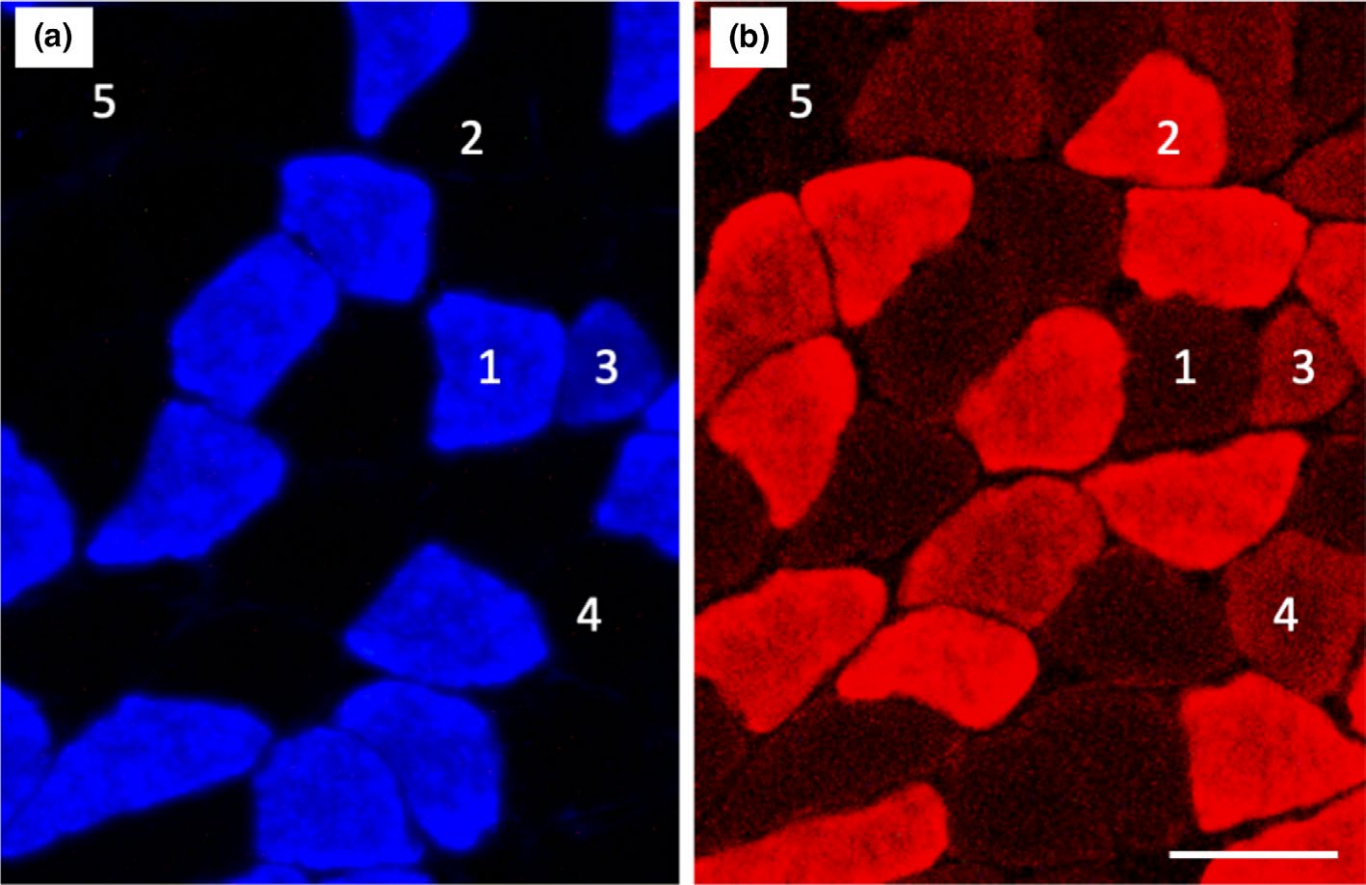
Representative immunohistochemical images of type I (a) and type IIA (b) muscle fibers. 1, type I fiber; 2, type IIA fiber; 3, type I + IIA fiber; 4, type IIAX fiber; 5, type IIB + IIX fiber. Horizontal bars represent 50 μm.

**FIGURE 5:**
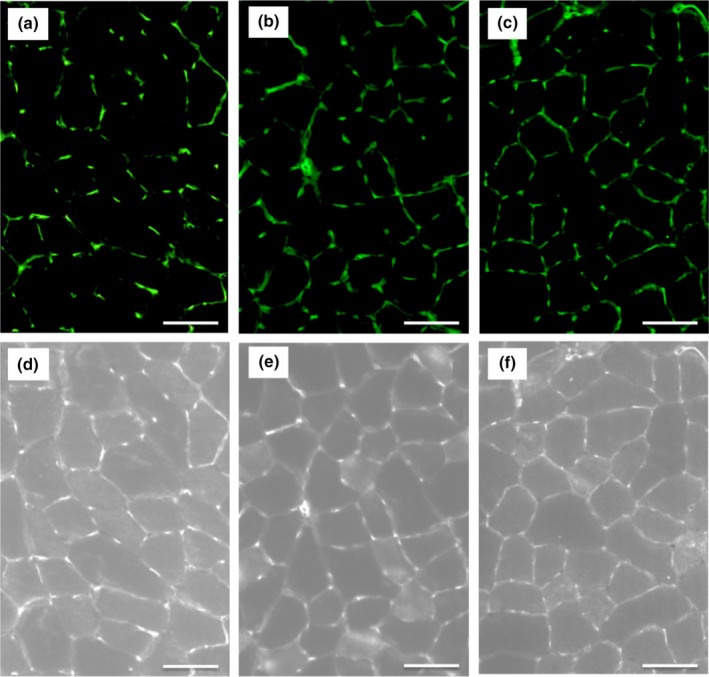
Representative images for capillary profiles (a–c) and muscle fiber profiles (d–f) of lateral red portion of gastrocnemius muscle (GrL) for SED (a and d), ET (b and e), and INT (c and f) groups. Horizontal bars represent 50 μm.

Representative immunofluorescent images for muscle fiber phenotypes and capillary profiles were shown in Figures 4 and 5, respectively. Histochemical examinations of capillary profiles and muscle fiber phenotypes were conducted as previously reported by the author with slight modifications (Suzuki, 2019b). Briefly, 12‐mm‐thick serial cross‐sections were obtained using a cryotome (MRS; Nihon Kouki Seisakusyo, Nagano, Japan) in a freezer set at −20°C from the mid‐belly portion of calf muscles. These sections were air‐dried, fixed with 100% ethanol at 4°C for 15 min, incubated in 0.1 M phosphate‐buffered saline (PBS) with 0.1% Triton X‐100, and washed in PBS. Sections were then blocked with 3% bovine serum albumin (010‐25783, Fujifilm‐Wako) at room temperature for 30 min, washed in PBS for 5 min, and incubated at 4°C overnight with a mixture of an anti‐type I myosin heavy chain (MHC) antibody (BA‐F8; mouse IgG2b, 1:80) and anti‐type IIA MHC antibody (SC‐71, mouse IgG1, 1:80) diluted with PBS. Sections were then reacted with Alexa Fluor 350‐labeled anti‐mouse IgG2b (1:500, A21140), Alexa Fluor 647‐labeled anti‐mouse IgG1 (1:500, A21240), and fluorescein‐labeled Griffonia simplicifolia lectin (GSL‐I) (1:100, [FL 1101; Vector Laboratories, Burlingame, CA, USA]) diluted with PBS at room temperature for 2 h. Sections were coverslipped with Fluoromount/Plus (K048; Diagnostic BioSystems, Pleasanton, CA, USA). Primary and secondary antibodies were purchased from the Developmental Studies Hybridoma Bank (University of Iowa) and Thermo Fisher Scientific (Tokyo, Japan), respectively. Fluorescent images of the incubated sections were observed using a microscope (Axio Observer; Carl Zeiss Japan, Tokyo, Japan) using an objective lens (Objective EC Plan‐Neofluar 20x/0.50 M27, #420350‐9900‐000, Carl Zeiss). All images were captured using Zen pro 2012 software (Carl Zeiss). Muscle fiber phenotypes were classified as type I (blue), type I + IIA (faint blue and faint red), type IIA (red), type IIAX (faint red), and type IIB + IIX (unstained, Figure 4). Fluorescent images were obtained from SOL, PL, the lateral (GrL) and medial (GrM) portions of Gr, and Gw. The negative control without primary antibodies was confirmed to show no fluorescent signal.

Nonoverlapping microscopic fields were selected at random from each tissue sample. The observer was blinded to the source (groups) of each slide during the measurements using a random number table.

### 2.7 Statistical analyses

#### 2.7.1 Procedures for significant testing

**FIGURE 6:**
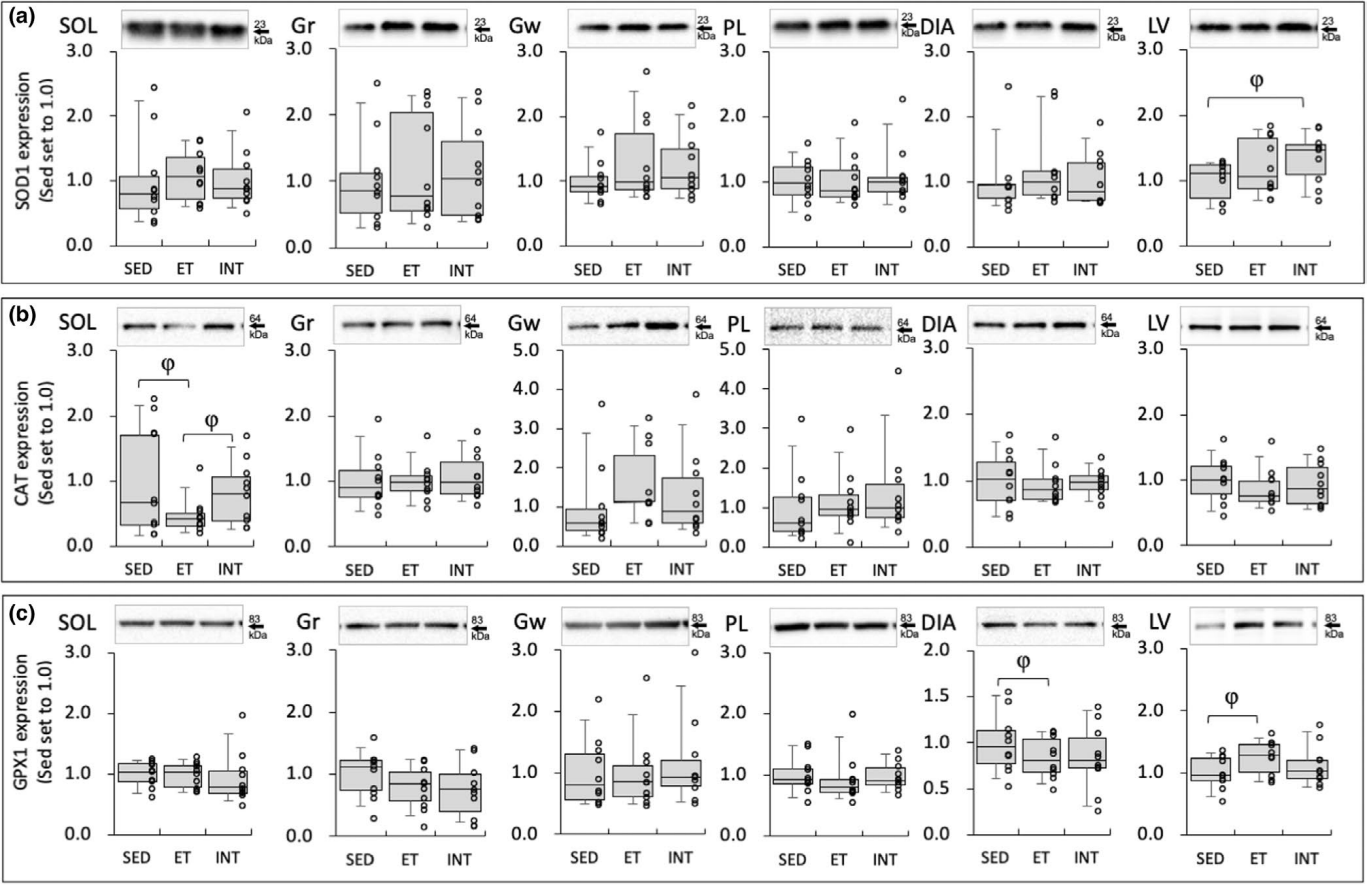
Protein levels for SOD1 (a), CAT (b), and GPX1 (c) at 48 hours after the last exercise session. The densities of the specific bands were normalized to the densities of all protein bands in each lane on the membrane (Gilda & Gomes, 2013). This normalizing procedure was confirmed to be superior to using β‐Actin as a loading control (Gilda & Gomes, 2013). Subsequently, the normalized densities of the bands were further normalized to the same sample that was run on every gel and transferred to every membrane, as reported by the author (Suzuki, 2021). Values are expressed as box and whisker plots with 5th, 25th, 50th, 75th and 95th percentile. Dots in the figure indicate data for each mouse. φ, The 95% confidential interval did not contain the mean value of target group for comparison.

**FIGURE 7:**
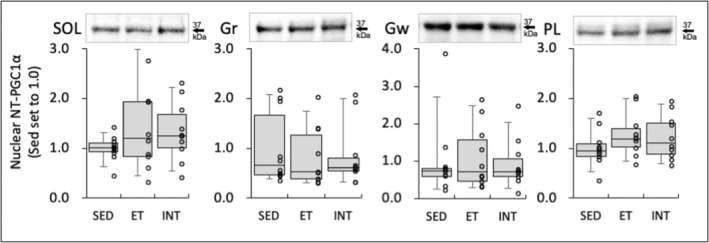
Protein levels for nuclear NT‐PGC1α protein at 48 h after the last exercise session. The densities of the specific bands were normalized to the densities of all protein bands in each lane on the membrane (Gilda & Gomes, 2013). This normalizing procedure was confirmed to be superior to using β‐actin as a loading control (Gilda & Gomes, 2013). Subsequently, the normalized densities of the bands were further normalized to the same sample that was run on every gel and transferred to every membrane, as reported by the author (Suzuki, 2021). Values are expressed as box and whisker plots with 5th, 25th, 50th, 75th, and 95th percentile. Dots in the figure indicate data for each mouse.

The statement on *p* values by the American Statistical Association (Wasserstein & Lazar, 2016) highlighted the widespread misuse and misunderstandings surrounding *p* values derived from null hypothesis significance testing (NHST). It also suggested alternative approaches, such as Bayes factors. Numerous articles have pointed out the limitations of NHST (e.g., Amrhein et al., 2019; Hentschke & Stüttgen, 2011; Nakagawa & Cuthill, 2007; Smith, 2018). It has been reported that Bayesian hypothesis testing, specifically using Bayes factors in the public domain JASP program, serves as a replacement for NHST in most situations (Kelter, 2020). While NHST can only reject the null hypothesis, the Bayes factor can provide evidence for both the null and alternative hypotheses, making hypothesis confirmation possible (Kelter, 2020). Therefore, this study employed Bayesian data analysis for statistical significance testing. All statistical analyses were performed using JASP (version 0.19.3). Differences between groups were examined using Bayesian ANOVA data analysis. If the Bayes factor (BF, specifically BF10 in JASP) was greater than 3.0, the study confirmed the difference as statistically significant. Additionally, when the 95% confidence interval (CI) values did not include the mean value of the target group for comparison, the differences were considered biologically important (Du Prel et al., 2009; Gardner & Altman, 1986) and were described in the text as substantial or considerable changes. Data are presented as individual plots along with box and whisker plots that include the 5th, 25th, 50th, 75th, and 95th percentiles in Figures 2, 3, 6, and 7. In tables, data are expressed as mean ± standard deviation (SD). Pearson's product–moment correlation coefficient was used to determine the correlations between two variables.

#### 2.7.2 Correlations

**FIGURE 8:**
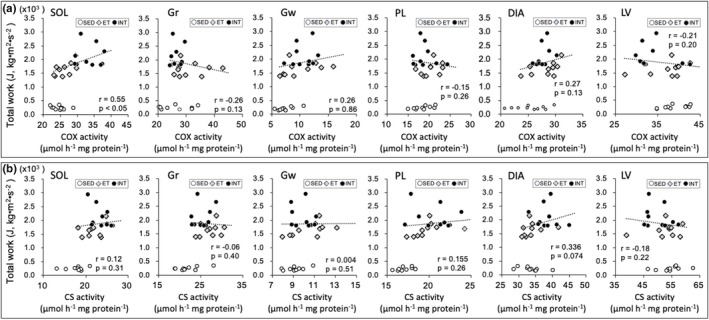
Scatter plots of COX (a) and CS (b) activity values against maximal endurance capacity. A correlation was calculated using data from ET and INT groups, because the present study focused on the difference between these two groups. Dots in the figure indicate data for each mouse.

**FIGURE 9:**
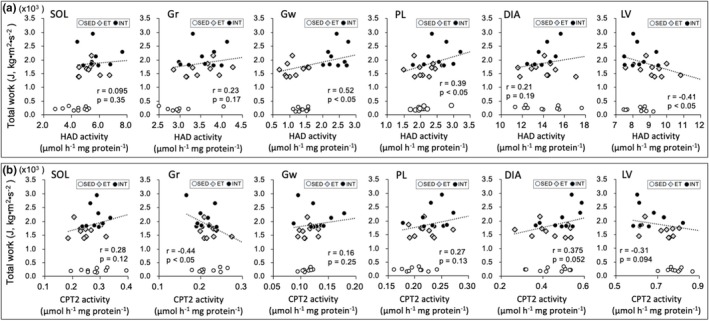
Scatter plots of HAD (a) and CPT2 (b) activity values against maximal endurance capacity. A correlation was calculated using data from ET and INT groups, because the present study focused on the difference between these two groups. Dots in the figure indicate data for each mouse.

**FIGURE 10:**
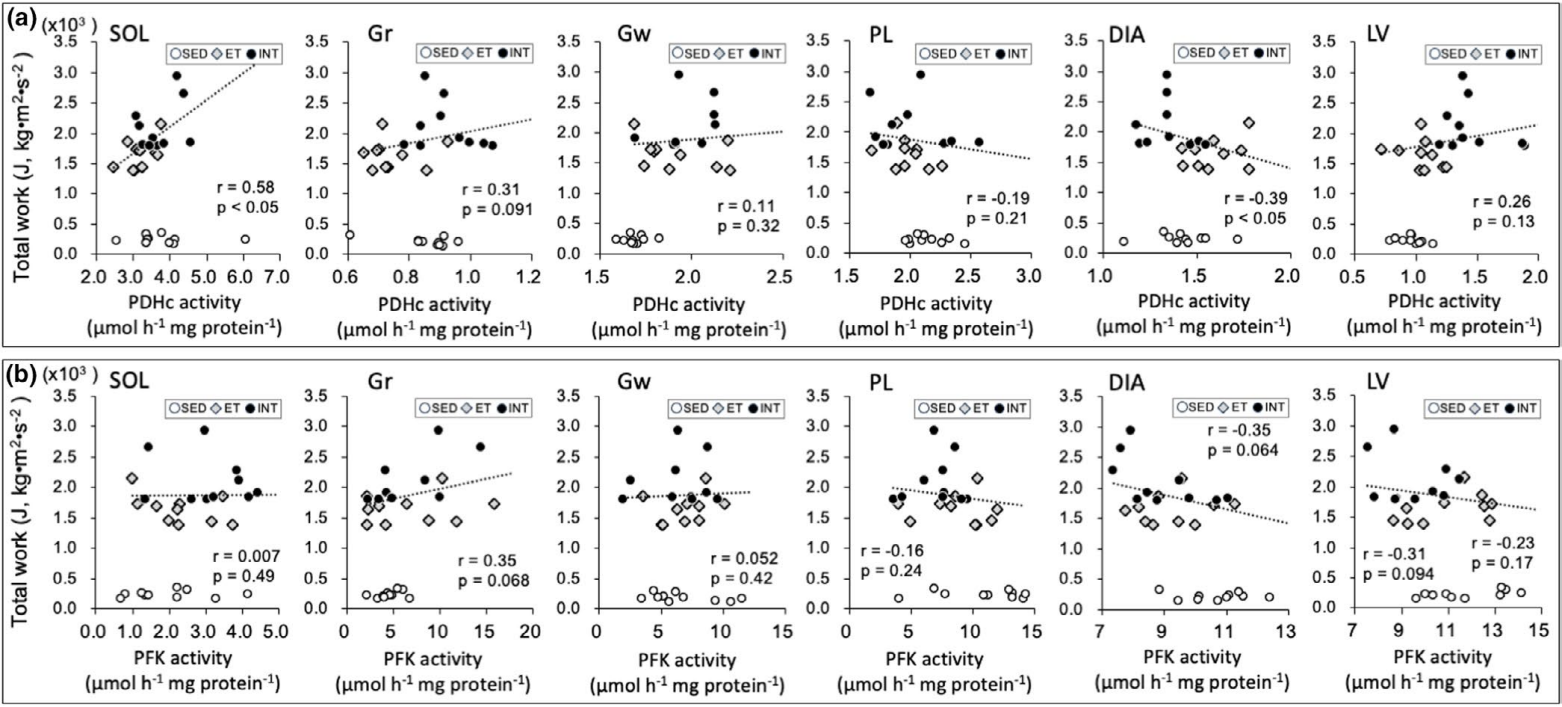
Scatter plots of PDHc (a) and PFK (b) activity values against maximal endurance capacity. A correlation was calculated using data from ET and INT groups, because the present study focused on the difference between these two groups. Dots in the figure indicate data for each mouse.

**FIGURE 11:**
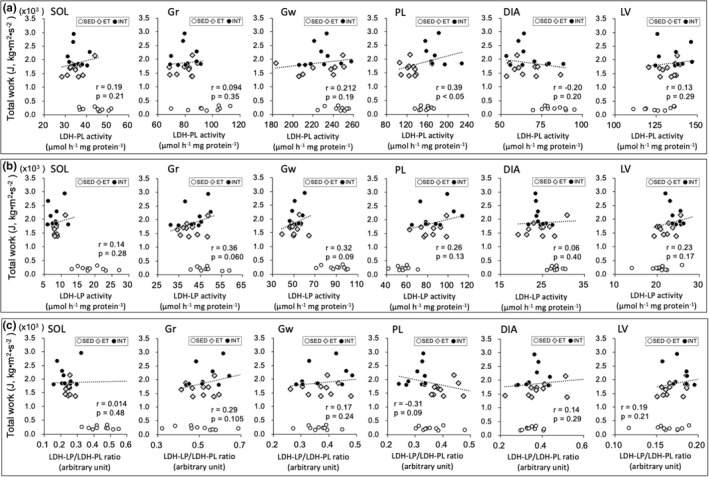
Scatter plots of LDH‐PL (a), LDH‐LP (b), and LDH‐LP/LDH‐PL ratio (c) values against maximal endurance capacity. A correlation was calculated using data from ET and INT groups, because the present study focused on the difference between these two groups. Dots in the figure indicate data for each mouse.

**FIGURE 12:**
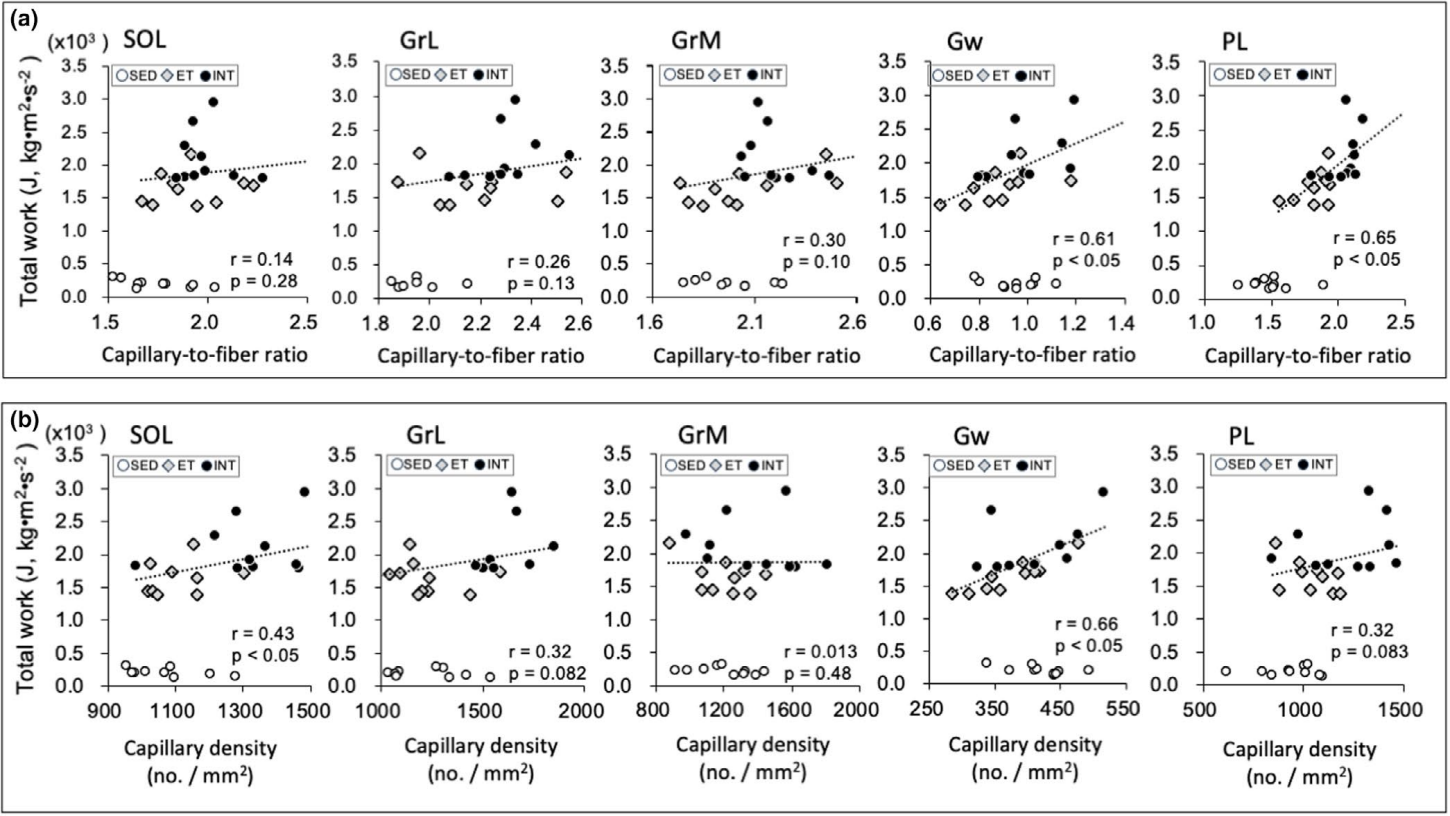
Scatter plots of capillary‐to‐fiber ratio (a) and capillary density (b) values against maximal endurance capacity. A correlation was calculated using data from ET and INT groups, because the present study focused on the difference between these two groups. Dots in the figure indicate data for each mouse.

Scatter plots of enzyme activity values (Figures 8, 9, 10, 11) and capillary profiles (Figure 12) were shown for all three groups against maximal endurance capacity. However, a correlation was calculated using data from the ET and INT groups, as this study focused on the differences between these two groups.

## 3 RESULTS

### 3.1 Body mass and maximal exercise capacity

After treadmill training, the body weights of the ET and INT groups were significantly lower than those of the Sed group (BF ≥ 3.0, Table 1). Following 4 weeks of treadmill training with or without hyperoxic exposure, the organ mass‐to‐body mass ratio showed significant increases in both the ET and INT groups across all observed tissues (BF ≥ 3.0).

**TABLE 1:** Body and organ mass values.

	SED (*n* = 10)	ET (*n* = 10)	INT (*n* = 10)
Body mass (g)			
After voluntary wheel run	38.9 ± 2.6	36.8 ± 1.6^§^	36.1 ± 1.6^§,ψ^
	[35.2–43.0]	[34.5–39.3]	[33.9–38.7]
After treadmill training	42.4 ± 2.7	39.3 ± 1.3^§,ψ,ω^	38.7 ± 1.5^§,δ,ω^
	[39.1–47.2]	[37.2–41.4]	[36.7–42.1]
Organ mass (mg)			
Soleus	9.60 ± 1.1	11.40 ± 1.4^§,ψ^	10.80 ± 1.30^§^
	[7.6–11.0]	[9.4–13.9]	[8.2–12.1]
Plantaris	22.2 ± 2.1	23.6 ± 2.3	23.4 ± 2.4
	[18.6–25.3]	[20.1–26.6]	[20.5–27.8]
Gastrocnemius	175.3 ± 16.4	177.4 ± 8.6	179.2 ± 11.6
	[159.0–199.9]	[159.6–189.4]	[160.3–203.4]
Whole heart	168.4 ± 6.9	184.0 ± 15.7^§,ψ^	186.5 ± 15.4^§,δ^
	[157.8–177.9]	[163.4–204.9]	[166.6–214.6]
Left ventricle	120.0 ± 11.7	135.2 ± 8.2^§,δ^	132.7 ± 11.4^§^
	[108.3–149.8]	[120.5–144.2]	[119.3–156.4]
Organ mass‐to‐body mass ratio (mg g^−1^)			
Soleus	0.23 ± 0.03	0.29 ± 0.04^§,ε^	0.28 ± 0.03^§,δ^
	[0.17–0.26]	[0.24–0.36]	[0.21–0.31]
Plantaris	0.53 ± 0.04	0.60 ± 0.06^§,δ^	0.61 ± 0.06^§,δ^
	[0.46–0.60]	[0.51–0.69]	[0.52–0.71]
Gastrocnemius	4.13 ± 0.17	4.51 ± 0.29^§,δ^	4.63 ± 0.2^§,ε^
	[3.9–4.4]	[3.9–4.8]	[4.4–5.0]
Whole heart	3.98 ± 0.18	4.68 ± 0.31^§,ε^	4.81 ± 0.29^§,ε^
	[3.7–4.2]	[4.3–5.2]	[4.4–5.3]
Left ventricle	2.84 ± 0.3	3.44 ± 0.14^§,ε^	3.42 ± 0.19^§,ε^
	[2.4–3.6]	[3.2–3.6]	[3.1–3.7]

*Note*: Values are presented as mean ± SD with range in brackets. ψ, δ, and ε, Τhe difference of mean values was significantly different from the SED group at BF ≥3.0, ≥10, and ≥30. ω, Τhe difference of mean values was significantly different from the pre‐treadmill training at BF ≥30. §, The 95% confidence interval did not contain the mean value of the SED group.

After 7 weeks of voluntary wheel running, total work values, obtained at “Pre‐testing” showing in Figure 1a, in the training group were significantly greater, by 7.4‐fold (BF ≥ 30, Figure 3a), than those in the SED group. After 4 weeks of hybrid training, total work values in the ET group did not significantly increase (1.01‐fold, BF = 0.315). In contrast, the INT group experienced a significant increase in total work values after hybrid training (1.3‐fold, BF = 18.3, Figure 3b). Additionally, total work values were significantly greater in the INT group compared to the ET group (1.3‐fold, BF = 6.3). Overall, exercise training under short‐duration intermittent hyperoxia had additive effects on improving endurance exercise capacity in well‐trained mice.

### 3.2 Enzyme activity

In SOL, COX levels were significantly higher in the INT group compared to the SED (1.4‐fold) and ET (1.3‐fold) groups (BF ≥ 30, Table 2) and showed a significant correlation with total work values (*r* = 0.55, *p* < 0.05, Figure 8a). In LV, COX values in the INT group were significantly and substantially lower than those in the SED group (BF ≥ 3.0) and ET group (CI: 0.82–0.99). In Gr, COX levels were significantly lower in the INT than in the ET group (BF = 3.6). CS activity levels in DIA were significantly higher in the INT group compared to the SED (BF = 7.8) and ET (BF = 7.1) groups (Table 2). In SOL, COX values were significantly correlated with total work values (*r* = 0.55, *p* < 0.05, Figure 8a). CS levels in SOL and PL showed significantly higher values in the ET (BF ≥ 10) and INT (BF ≥ 30) groups compared to the SED group. Thus, in the INT group, COX levels in the highly oxidative SOL muscle were enhanced, while CS levels in the respiratory muscle were increased.

**TABLE 2:** Enzyme activity values (μmol h^−1^ mg protein^−1^).

		SED (*n* = 10)	ET (*n* = 10)	INT (*n* = 10)		
COX	SOL	24.4 ± 2.0	26.1 ± 2.5	33.5 ± 3.5^εθ§¶^		
	Gr	25.8 ± 5.5	31.6 ± 5.7	26.6 ± 1.8^κ¶^		
	Gw	7.3 ± 1.5	10.0 ± 3.0^§^	10.6 ± 1.9^ε§^		
	PL	18.4 ± 2.2	20.3 ± 3.0^§^	18.8 ± 2.6		
	DIA	25.4 ± 2.9	28.2 ± 2.5^§^	27.8 ± 1.3^§^		
	LV	38.9 ± 3.1	37.6 ± 4.4	34.0 ± 4.3^ψ§¶^		
CS	SOL	17.9 ± 2.2	21.9 ± 2.3^λ§^	23.7 ± 2.1^ε§^		
	Gr	22.0 ± 2.7	27.3 ± 2.2^ε§^	25.4 ± 2.0^ψ§¶^		
	Gw	9.1 ± 0.86	10.5 ± 1.7^§^	10.0 ± 0.93^§^		
	PL	17.3 ± 0.94	19.8 ± 1.9^δ§^	20.7 ± 2.2^θ§^		
	DIA	33.9 ± 3.2	33.9 ± 3.2	38.4 ± 3.3^ψκ§¶^		
	LV	55.0 ± 4.4	52.8 ± 5.6	50.2 ± 4.3_§_		
HAD	SOL	4.4 ± 0.82	5.3 ± 0.71^ψ§^	5.7 ± 0.92^δ§^		
	Gr	2.9 ± 0.51	3.5 ± 0.43^ψ§^	3.5 ± 0.40^ψ§^		
	Gw	1.0 ± 0.20	1.1 ± 0.30	2.5 ± 0.43^εθ§¶^		
	PL	2.1 ± 0.33	2.1 ± 0.30	2.5 ± 0.43^ψκ§¶^		
	DIA	14.0 ± 1.96	13.9 ± 1.64	13.9 ± 1.05		
	LV	8.3 ± 0.58	9.2 ± 0.88^ψ§^	8.3 ± 0.69^κ¶^		
CPT2	SOL	0.28 ± 0.06	0.25 ± 0.04^§^	0.29 ± 0.03^¶^		
	Gr	0.22 ± 0.03	0.23 ± 0.02	0.21 ± 0.02		
	Gw	0.11 ± 0.01	0.11 ± 0.01	0.13 ± 0.03^§^		
	PL	0.20 ± 0.02	0.22 ± 0.02^§^	0.23 ± 0.03^ψ§^		
	DIA	0.44 ± 0.08	0.44 ± 0.09	0.50 ± 0.07^§¶^		
	LV	0.78 ± 0.04	0.76 ± 0.11	0.66 ± 0.07^ε§¶^		
PDHc	SOL	3.78 ± 0.92	3.18 ± 0.40^§^	3.72 ± 0.52^¶^		
	Gr	0.86 ± 0.10	0.75 ± 0.08^ψ§^	0.92 ± 0.10^θ¶^		
	Gw	1.69 ± 0.06	1.92 ± 0.20^δ§^	1.97 ± 0.14^θ§^		
	PL	2.13 ± 0.16	1.99 ± 0.16^§^	2.01 ± 0.30		
	DIA	1.43 ± 0.16	1.59 ± 0.14^§^	1.35 ± 0.13^θ¶^		
	LV	0.96 ± 0.10	1.04 ± 0.16	1.46 ± 0.24^εθ§¶^		
PFK	SOL	2.0 ± 1.1	2.3 ± 0.92	3.1 ± 1.1^§¶^		
	Gr	4.7 ± 1.3	6.7 ± 4.8	6.5 ± 3.9		
	Gw	7.0 ± 2.8	6.9 ± 1.9	6.5 ± 2.5		
	PL	10.8 ± 3.5	8.8 ± 2.7^§^	7.1 ± 2.0^ψ§¶^		
	DIA	10.7 ± 1.1	9.3 ± 1.1^§δ^	8.9 ± 1.3^θ§^		
	LV	11.9 ± 1.6	11.0 ± 1.6	9.5 ± 1.4^δ§¶^		
LDH‐PL	SOL	44.5 ± 5.5	34.3 ± 4.3^ε§^	35.8 ± 3.5^ε§^		
	Gr	99.0 ± 15.9	80.0 ± 7.3^δ§^	80.3 ± 7.6^δ§^		
	Gw	245.8 ± 10.9	224.4 ± 22.7^ψ§^	229.2 ± 15.7^ψ§^		
	PL	155.0 ± 11.5	124.8 ± 11.3^ε§^	176.9 ± 20.9^ψθ§^		
	DIA	82.1 ± 7.7	65.3 ± 10.0^ε§^	67.9 ± 11.1^δ§^		
	LV	125.6 ± 8.6	131.8 ± 5.6^§^	136.4 ± 8.1^ψ§^		
LDH‐LP	SOL	19.3 ± 4.2	8.6 ± 1.0^ε§^	8.4 ± 2.0^ε§^		
	Gr	48.5 ± 5.5	40.6 ± 5.0^δ§^	42.1 ± 6.0^§^		
	Gw	89.5 ± 9.8	83.2 ± 11.5	86.2 ± 16.9		
	PL	56.5 ± 7.7	50.1 ± 6.6	54.0 ± 4.5^¶^		
	DIA	28.0 ± 1.3	24.5 ± 3.4^ψ§^	24.3 ± 1.6^ε§^		
	LV	20.7 ± 3.0	22.1 ± 2.0	24.6 ± 1.4^δκ§¶^		
LDH‐LP/‐PL ratio	SOL	0.43 ± 0.07	0.25 ± 0.02^ε§^	0.23 ± 0.05^ε§^		
	Gr	0.50 ± 0.10	0.51 ± 0.06	0.53 ± 0.07		
	Gw	0.37 ± 0.05	0.37 ± 0.06	0.38 ± 0.07		
	PL	0.37 ± 0.05	0.40 ± 0.04^§^	0.31 ± 0.03^ψθ§¶^		
	DIA	0.34 ± 0.04	0.39 ± 0.09	0.37 ± 0.06		
	LV	0.16 ± 0.02	0.17 ± 0.01	0.18 ± 0.01^§¶^		

*Note*: Values are presented as mean ± SD. ψ, δ, and ε, Τhe difference of mean values was significantly different from the SED group at BF ≥3.0, ≥10, and ≥30. κ, λ, and θ, Τhe difference of mean values was significantly different from the ET group at BF ≥3.0, ≥10, and ≥30. § and ¶, The 95% confidence interval did not contain the mean value of the SED and ET groups, respectively.

HAD activity values in SOL and Gr were significantly higher in the ET and INT groups compared to the SED group (BF ≥ 3.0, Table 2). In Gw (BF ≥ 30) and PL (BF = 5.4), HAD values in the INT group were significantly higher than those in the ET group. Additionally, HAD values were significantly correlated with total work values in Gw (r = 0.52, *p* < 0.05) and in PL (*r* = 0.39, p < 0.05, Figure 9a). CPT2 values were substantially higher in the INT group compared to the ET group in SOL (CI: 1.07–1.24) and DIA (CI: 1.02–1.26, Table 2). Conversely, in LV, CPT2 levels were substantially lower in the INT group than in the ET group (CI: 0.80–0.93). CPT2 values in Gr showed a significant negative correlation with total work values (*r* = −0.44, *p* < 0.05, Figure 9b). Thus, enzyme activity levels related to fatty acid metabolism were enhanced markedly in the INT group in predominantly glycolytic muscle regions.

In Gr and LV, PDHc activity levels were significantly higher in the INT group compared to the ET group (BF ≥ 30, Table 2). Moreover, in SOL, PDHc levels showed substantially higher values in the INT group compared to the ET group (CI: 1.05–1.28). PDHc values in SOL showed a significant positive correlation with total work values (*r* = 0.58, *p* < 0.05, Figure 10a). In DIA, however, PDHc levels were significantly lower in the INT group than in the ET group. Moreover, PDHc levels were negatively correlated with maximal work values in DIA (*r* = −0.39, *p* < 0.05, Figure 10a). Consequently, the utilization of pyruvic acid was facilitated in the primarily oxidative muscle portion and LV, but was reduced in DIA.

PFK activity levels in SOL were substantially higher in the INT group (CI: 1.02–1.69) group compared to the ET group (Table 2). In contrast, PFK values were substantially lower in the INT group compared to the ET group in both PL (CI: 0.65–0.97) and LV (CI: 0.77–0.95). Moreover, PFK levels in DIA showed significantly lower values in both ET and INT groups than in the SED group (BF > 10). Thus, in the INT group, activity levels of the rate‐limiting enzyme for glycolysis were considerably increased in the highly oxidative muscle, while they were substantially decreased in glycolytic muscle and the heart.

LDH‐PL activity levels in PL were significantly higher in the INT group than in the ET group (BF ≥ 30, Table 2). LDH‐PL values in PL showed a significant positive correlation with total work values (*r* = 0.39, *p* < 0.05, Figure 11a). Additionally, LDH‐LP levels in PL were substantially higher in the INT group compared to the ET group (CI: 1.01–1.14, Figure 11b). In LV, LDH‐LP levels were also significantly higher in the INT group than in the ET group (BF = 8.3). The LDH‐LP/PL ratio values in PL were significantly lower in the INT group than in the ET group (BF ≥ 30, Figure 11c). In LV, the LDH‐LP/PL ratio levels were substantially higher in the INT group than in the ET group (CI: 1.03–1.13). Thus, in the INT group, lactic acid production was likely enhanced in glycolytic muscle, while its utilization was increased in the heart.

### 3.3 Protein levels

Protein expression levels were determined by analyzing tissue samples collected 48 h after the final performance test.

Protein levels of SOD1 in LV were substantially higher in the INT group than in the SED group (1.3‐fold, CI: 1.07–1.62, Figure 6a). In SOL, CAT expression levels in the INT group were comparable to that of the SED (Figure 6b). However, CAT levels in the ET group were notably lower than those in the SED group (0.46‐fold, CI: 0.26–0.66) and the INT (0.57‐fold, CI: 0.32–0.82) groups. Additionally, CAT expression levels in SOL were positively correlated with total work values (*r* = 0.40, *p* < 0.05). In the ET group, GPX1 expression levels were substantially higher in LV (1.2‐fold, CI: 1.05–1.45) and were lower in DIA (0.84‐fold, CI: 0.68–0.99) compared to the SED group (Figure 6c).

Nuclear NT‐PGC1α levels in calf muscles were not changed in the present study (Figure 7).

### 3.4 Muscle fiber‐type composition

The proportion of type I and IIA fibers in SOL was significantly higher in the ET group compared to the SED group (BF ≥ 3.0, Table 3). Additionally, the proportion of hybrid type I + IIA fibers in SOL was significantly greater in the INT group than in the SED group (BF ≥ 3.0). In PL, the proportion of type I fibers was significantly higher in the INT group than in the ET group (BF ≥ 3.0). A positive correlation was observed between the proportion of type I fibers and total work value in PL (*r* = 0.46, *p* = 0.02). The proportion of type IIA fibers in PL was significantly higher in the ET (BF ≥ 10) and INT (BF ≥ 30) groups compared to the SED group. Additionally, in PL, the proportion of type IIB + IIX was substantially lower in the INT group than in the SED (CI: 0.51–0.24) and ET groups (CI: 0.87–0.42). In GrL, the proportion of type IIA fibers was significantly higher in the ET (BF ≥ 30) and INT (BF ≥ 3.0) groups than in the SED group. Thus, exercise under intermittent hyperoxia significantly increased and substantially reduced the proportion of type I and type IIB + IIX fibers, respectively, in fast‐twitch muscle.

**TABLE 3:** Fiber type composition values (%).

		SED (*n* = 10)	ET (*n* = 10)	INT (*n* = 10)
SOL	I	45.7 ± 5.3	55.2 ± 7.9^ψ,§^	51.0 ± 6.4^§^
	IIA	54.1 ± 5.4	44.2 ± 8.0^ψ^	47.8 ± 6.9
	I + IIA	0.16 ± 0.34	0.57 ± 0.57	1.16 ± 1.15^ψ^
PL	I	4.51 ± 3.0	2.48 ± 1.16^§^	5.66 ± 3.13^κ,¶^
	IIA	44.3 ± 9.1	60.9 ± 10.6^δ,§^	59.0 ± 5.0^ε,§^
	I + IIA	0.06 ± 0.14	0.24 ± 0.30	0.12 ± 0.16^¶^
	IIAX	17.30 ± 10.3	16.7 ± 4.4	22.5 ± 5.9^§,¶^
	IIB + IIX	33.8 ± 15.7	19.7 ± 10.3^§^	12.7 ± 6.3^ε,§,¶^
GrL	I	18.0 ± 4.1	17.5 ± 4.0	17.1 ± 3.1
	IIA	44.5 ± 3.7	51.3 ± 3.5^ε,§^	51.1 ± 5.7^ψ,§^
	I + IIA	0.07 ± 0.24	0.07 ± 0.23	0.12 ± 0.23
	IIAX	9.38 ± 4.8	10.2 ± 6.5	10.9 ± 5.9
	IIB + IIX	28.1 ± 9.1	20.9 ± 8.8	20.8 ± 9.9
GrM	I	36.6 ± 6.0	40.2 ± 5.1^§^	36.3 ± 6.7
	IIA	49.4 ± 9.2	55.8 ± 6.2^§^	59.4 ± 6.2^ψ,§^
	I + IIA	0.12 ± 0.24	0.10 ± 0.22	0.28 ± 0.30
	IIAX	13.9 ± 11.8	3.85 ± 7.2	4.10 ± 5.6
GW	IIB + IIX	100	100	100

*Note*: Values are presented as mean ± SD. ψ, δ, and ε, Τhe difference of mean values was significantly different from the SED group at BF ≥3.0, ≥10, and ≥30. κ, λ, and θ, Τhe difference of mean values was significantly different from the ET group at BF ≥3.0, ≥ 10, and ≥30. § and ¶, The 95% confidence interval did not contain the mean value of the SED and ET groups, respectively.

### 3.5 Capillarization

Values of the capillary‐to‐fiber ratio (C/F) in SOL (BF ≥ 3.0) and PL (BF ≥ 30) were significantly higher in the INT group compared to the ET group (Table 4). The C/F values were substantially greater in the INT group than in the ET group in GrL (CI: 1.002–1.10) and GrM (CI: 1.03–1.12). C/F values showed a positively significant correlation with total work values in Gw (*r* = 0.61, *p* < 0.05) and PL (*r* = 0.65, *p* < 0.05, Figure 12a). Capillary density values in GrL were significantly higher in the INT group than in the ET group (BF ≥ 3.0). Furthermore, capillary density values in SOL (CI: 1.04–1.25) and PL (CI: 1.03–1.32) were considerably greater in the INT group than in the ET group. A significant positive correlation was observed between capillary density values and total work values in SOL (*r* = 0.43, *p* < 0.05) and Gw (*r* = 0.66, *p* < 0.05, Figure 12b). Thus, exercise under short‐duration intermittent hyperoxia facilitates exercise‐induced capillary growth in the hindlimb of highly trained mice.

**TABLE 4:** Capillary‐to‐fiber ratio and capillary density values.

	SED (*n* = 10)	ET (*n* = 10)	INT (*n* = 10)
Capillary‐to‐fiber ratio			
SOL	1.75 ± 0.17	1.91 ± 0.19^§^	1.99 ± 0.13^κ^
GrL	1.89 ± 0.14	2.18 ± 0.21^δ§^	2.30 ± 0.13^εk§¶^
GrM	1.94 ± 0.21	2.04 ± 0.26	2.20 ± 0.14^ψ§¶^
Gw	0.95 ± 0.11	0.88 ± 0.15	0.98 ± 0.15
PL	1.50 ± 0.17	1.82 ± 0.13^§^	2.06 ± 0.11^εθ§¶^
Capillary density (number per mm^2^)			
SOL	1151.8 ± 258.5	1152.3 ± 160.3	1318.9 ± 147.9^§¶^
GrL	1227.0 ± 175.7	1231.2 ± 163.9	1538.5 ± 251.0^ψκ§¶^
GrM	1211.6 ± 172.7	1201.0 ± 165.3	1381.8 ± 270.8^§^
Gw	419.9 ± 42.7	372.8 ± 57.4	411.9 ± 63.5
PL	943.7 ± 151.0	1042.9 ± 115.0^§^	1226.5 ± 212.1^δ§¶^

*Note*: Values are presented as mean ± SD. ψ, δ, and ε, Τhe difference of mean values was significantly different from the SED group at BF ≥3.0, ≥10, and ≥30. κ, λ, and θ, Τhe difference of mean values was significantly different from the ET group at BF ≥3.0, ≥10, and ≥30. § and ¶, The 95% confidence interval did not contain the mean value of the SED and ET groups, respectively.

## 4 DISCUSSION

The body weight showed a significant increase after 4 weeks of exercise training, both with and without intermittent hyperoxia (Table 1). Thus, it is likely that the present hybrid exercise training under intermittent hyperoxia did not cause distress in well‐trained mice.

In the present study, 7 weeks of voluntary wheel training significantly increased endurance exercise performance (Figure 3b). Endurance performance in mice has been shown to improve after 4 (Kim et al., 2023; Wada et al., 2015) and 8 weeks (Bell et al., 2019) of voluntary wheel running. The present study demonstrated that hybrid exercise training, comprising endurance and interval exercise, combined with short‐duration intermittent hyperoxic exposure (referred to as INT training), had an additive effect on improving endurance exercise performance in well‐trained mice (Figure 3b).

Significant increases in enzyme activity levels compared to the ET group were observed for COX values in SOL, CS values in DIA, HAD values in Gw and PL, PDHc values in Gr and LV, LDH‐PL values in PL, and LDH‐LP values in LV (Table 2). In contrast, a significant decrease in activity levels was shown for the LDH‐LP/‐PL ratio in PL and PDHc levels in DIA (Table 2). Thus, the improvement in endurance performance after INT training is likely induced by these metabolic enzyme levels.

COX (complex IV) is the last step of the electron transport chain, receiving electrons from cytochrome *c* and irreversibly reducing oxygen to water (Kadenbach & Hüttemann, 2015). COX is the rate‐limiting enzyme of mitochondrial respiration and plays a crucial role in aerobic energy metabolism by controlling mitochondrial respiration (Ramzan et al., 2021). After 8 weeks of voluntary running, COX activity levels were shown to be unchanged in the gastrocnemius muscle (Bell et al., 2019). Thus, changes in COX levels in the present study presumably reflected the effects of ET or INT training. As mentioned above, COX levels in SOL showed drastically greater values than those in the ET group (by 1.3‐fold, Table 2) the levels also showed a significantly positive correlation with maximal work values (Figure 8a). However, COX activity levels in Gr were comparable to those in the SED group and were significantly lower than those in the ET group (Table 2). Unlike SOL, the Gr muscle portion contains fast‐twitch (type IIAX and IIB + IIX) muscle fibers (Table 3). Thus, the current INT training with hybrid exercise may stimulate glycogen usage in the Gr muscle portion, described as follows.

After INT training, glycogen usage is likely facilitated in Gr and LV, identified as a marked increase in PDHc levels compared to the ET group (Table 2). Moreover, in SOL, PDHc levels were positively correlated with total work values (Figure 10a). PDHc is a complex of three enzymes that converts pyruvate into acetyl‐CoA, which is then used in the citric acid cycle. This complex links the glycolytic pathway to the citric acid cycle. During cycling exercise, PDHc activation in the human vastus lateralis muscle was directly proportional to relative aerobic power output (% of maximal oxygen consumption) (Spriet & Heigenhauser, 2002). The increased PDHc activity levels observed after the present INT training likely allow individuals to perform exercises at higher intensities. In well‐trained mice, hybrid training as used in the present study failed to improve PDHc levels (Suzuki, 2019b). Therefore, the present INT training likely promotes glycogen utilization by upregulating PDHc activity levels, especially in Gr, SOL, and the heart in well‐trained mice. In rats, voluntary wheel running for 8 weeks did not change PDHc activity levels in skeletal muscle (Nakai et al., 1999). Thus, the changes in PDHc activity levels observed in the present study likely reflect the effects of hybrid training with or without INT. In contrast, in DIA, a negative correlation was observed between PDHc activity levels and total work values (*r* = −0.39, *p* < 0.05, Figure 10a), and CS and CPT2 activity levels were significantly and substantially, respectively, higher in the INT than in the ET group (Table 2). Thus, it is suspected that fatty acids, rather than pyruvic acid, were used by the DIA as fuel during INT exercise.

In this study, HAD activity levels in Gw and PL muscles significantly improved after INT training (Table 2), and these levels were positively correlated with total work values (Figure 9a). In mice, Gw and PL muscle regions comprise approximately 40% and 5%, respectively, of the calf cross‐sectional area at the mid‐belly (Suzuki, 2019b). Although blood flow to Gw was shown to be unchanged during exercise in untrained rats (Delp et al., 1989), blood flow to the fast‐twitch white region of the calf muscle increased by 40%–50% after exercise training (Mackie & Terjung, 1983). Thus, facilitated fatty acid utilization in Gw and PL probably helps to enhance endurance performance. After 6 weeks of voluntary running in rats, HAD activity levels did not significantly change in Gr (Laye et al., 2009). Thus, the changes in HAD activity levels observed in the present study likely reflect the effects of hybrid training with or without INT. In contrast, in Gr, a negative correlation was observed between CPT2 activity levels and total work values (*r* = −0.44, *p* < 0.05, Figure 9b), and PDHc activity levels were significantly higher in the INT than in the ET group (Table 2). Thus, pyruvic acid, rather than fatty acid, appears to be used by the Gr as fuel during INT exercise.

In a previous study by the author (Suzuki, 2024), mice in the INT training group ran under intermittent hyperoxia (30%) for 3 days per week over 4 weeks; that is, the INT training was conducted 12 times in total. In these mice, nuclear NT‐PGC1α protein levels were considerably higher (1.6‐fold) in the INT group than in the ET group in SOL when the muscle was excised 48 h after the last exercise bout (Suzuki, 2024). PGC1α protein levels were significantly higher (approximately 2.3‐fold) after 7.5 weeks of endurance training (5 days per week) in rats (Taylor et al., 2005). In their study, muscle samples were collected at 20–24 h after the last exercise bout. In humans, acute exercise to exhaustion significantly enhanced PGC1α protein levels until 24 h but not at 52 h post exercise (Mathai et al., 2008). In the present study, nuclear NT‐PGC1α protein levels 48 h following the last exercise bout did not show any substantial change in all muscles examined (Figure 7). Mice in the present training groups were subjected to voluntary exercise for 49 days, followed by INT training for 24 days (6 days per week for 4 weeks) in total. Thus, muscle metabolic adaptation may have been nearly completed in the early stage of the 4‐week INT training; consequently, in the later stage, the stimulus that induces PGC1α upregulation may be blunted. Experiments that observe time‐course changes in muscle adaptive responses during INT training are likely to clarify these questions.

The LDH enzyme exists in a tetramer formation and has five LDH isomeric forms comprised of different ratios of the two subunits, M and H (Markert, 1963), which are encoded by the LDHA and LDHB genes, respectively (Li, 1989). The H isomer is predominantly found in the myocardium, where it converts lactate to pyruvate in aerobic conditions. Conversely, the M isomer is abundant in skeletal muscles and converts pyruvate to lactate under anaerobic conditions. Unlike the LDHB gene, the LDHA gene contains hypoxia recognition sites in its promoter sequence, making it responsive to HIF1α (Semenza et al., 1994). Consequently, the LDHA transcription is upregulated by acute hypoxia (Firth et al., 1995), while LDHB generally shows no significant response to hypoxia (Osis et al., 2021). In the present study, the LDH‐LP/‐PL ratio was observed, potentially indicating the H/M isozyme ratio. The present INT training decreased the LDH‐LP/‐PL ratio in PL (Table 2), suggesting that this training may increase the expression of the LDH‐M isozyme, facilitating the conversion of pyruvate to lactate. In contrast, in LV, both LDH‐LP activity levels and the LDH‐LP/‐PL ratio increased significantly and considerably, respectively, after INT training. In LV, a negative correlation between HAD activity levels and total work values was observed (*r* = −0.41, *p* < 0.05, Figure 9a), and HAD and CPT2 activity levels were significantly and substantially, respectively, lower in the INT than in the ET group (Table 2). Thus, it is suspected that lactate produced in the working muscle is used by the heart as fuel, while fatty acid utilization is reduced during INT exercise.

In the present study, 48 h after the last exercise bout, CAT protein levels in SOL were considerably reduced in the ET group compared to the SED (0.46‐fold, CI: 0.25–0.66) and INT (0.57‐fold, CI: 0.32–0.82, Figure 6b) groups. However, CAT levels in the INT group showed a comparable level to the SED group. Additionally, CAT protein levels in SOL showed a significant positive correlation with maximal work values (*r* = 0.40, *p* = 0.042). Transgenic overexpression of CAT in mitochondria probably improved endurance performance (Li et al., 2009), but in another report, it did not affect endurance exercise performance (Rao et al., 2018). CAT is an antioxidant enzyme that converts hydrogen peroxide into molecular oxygen and water. To remove H_2_O_2_ from various regions of the cell, CAT is located in several cellular compartments, including peroxisomes, the cytosol, and mitochondria. There is limited evidence to suggest that exercise training leads to an increase in CAT activity in skeletal muscle (Higuchi et al., 1985; Powers et al., 1999). Numerous studies have indicated that exercise training may reduce CAT activity in skeletal muscles (Alessio & Goldfarb, 1988; Laughlin et al., 1990). However, the reason for the decrease in CAT activity following endurance training in muscles remains unclear. After 5 days of endurance training, CAT activity levels were significantly increased and were significantly correlated with endurance performance in DIA assessed by fatigue index in vitro (Vincent et al., 2000). Thus, retained CAT levels after INT training in SOL probably contribute to promoting exercise‐induced adaptation, thereby facilitating endurance exercise performance.

In PL, INT training facilitated an increase in the proportion of type I fibers (Table 3). A positive correlation was observed between the proportion of type I fibers and total work value in PL (*r* = 0.46, *p* = 0.02). After 4 weeks of voluntary wheel running in mice, the proportion of type I fibers was comparable to that in the sedentary mice in PL (Waters et al., 2004). Moreover, after 10 weeks of wheel running, the expression of type I myosin heavy chain did not change in lower leg muscles (Schmitt et al., 2020). Thus, changes in the proportion of type I fibers observed in PL likely reflect the effects of hybrid training with INT. An increase in type I fibers has been shown to promote endurance performance, fatigue resistance, and oxidative metabolism (Zierath & Hawley, 2004). Additionally, in the present study, maximal work values were significantly correlated with improvements in capillary profiles in SOL, Gw, and PL (Figure 12).

It has been noted that muscle capillarization is probably one of the limiting factors for exercise performance (Hellsten & Gliemann, 2024). Capillary growth likely contributes to enhanced oxygen transport to muscle tissue, thereby improving endurance performance. Using well‐trained mice, as used in the present study, 4 weeks of hybrid training increased capillary supply compared to the SED group only in PL muscle (Suzuki, 2019b, 2021). Moreover, in these studies, intermittent hypoxic exposure (Suzuki, 2019b) or hyperbaric exposure (Suzuki, 2021) at rest did not cause a further increase in capillary supply. In the present study, capillary supply in both oxidative (SOL and GrM) and glycolytic (PL) muscle portions was enhanced by INT training (Table 4). After 4 weeks of voluntary wheel running in mice, the C/F ratio values were significantly increased in SOL (Wada et al., 2015) and PL (Wada et al., 2015; Waters et al., 2004). Thus, it is likely that muscle capillary profiles had already improved after the 7‐week wheel run. Muscle‐specific deletion of VEGF markedly reduced endurance performance and decreased capillary supply, despite significant increases in HAD, CS, and PFK activity levels in the gastrocnemius muscle (Olfert et al., 2009). These results suggest that endurance performance largely depends on muscle capillary supply. In the present study, the voluntarily trained mice were divided into ET and INT groups to match maximal exercise performance values (Figure 3a). Therefore, the capillary profiles in the ET and INT groups at the onset of treadmill training may have been at an equivalent level. Differences in capillary profiles between the ET and INT groups after treadmill training likely reflect the effects of intermittent hyperoxic intervention. Exercise‐induced capillary growth was shown to be predominantly induced by HIF1‐mediated upregulation of VEGF. Expression levels of VEGF were shown to be markedly increased in the early stage of exercise training but tended to be suppressed thereafter. In rats, VEGF protein levels were significantly increased at 3 days of endurance training but were comparable to the sedentary group at 13 weeks (Amaral et al., 2008). In humans, VEGF mRNA levels in response to exercise were diminished after 4 weeks of exercise training (Jensen et al., 2004). The present long‐term exercise training, that is, 7 weeks of voluntary running followed by 4 weeks of INT exercise, probably blunted VEGF expression after 48 hours of the last run. Thus, any evidence of factors other than an increased number of capillaries that induce angiogenesis may not be found.

## 5 CONCLUSION

In the present study, male mice were trained for 7 weeks through voluntary running, resulting in a 7.4‐fold increase in endurance performance. Thereafter, the trained mice were subjected to INT training, which involves exercise training combined with short‐duration intermittent hyperoxic intervention (30% O_2_) for 4 weeks (6 days per week) and offers additional benefits for improving endurance performance. INT training has been shown to enhance enzyme activity levels associated with oxidative phosphorylation in the soleus muscle and fatty acid metabolism in predominantly glycolytic Gw and PL muscles. In the Gr muscle portion, a negative correlation between CPT2 activity levels and total work values, along with increased PDHc activity levels, likely indicates that INT training promoted the use of pyruvic acid rather than fatty acids as fuel. According to the increased activity levels of PDHc and LDH‐LP, and the decreased activity levels of HAD, INT training may promote the utilization of pyruvic acid through its conversion from lactic acid, while reducing fatty acid utilization in the heart. Additionally, INT training kept catalase protein levels unchanged, while training without INT substantially decreased the levels in predominantly oxidative soleus muscle. These findings highlight the potential advantages of INT training as a strategy for enhancing endurance performance in well‐trained mice.

## AUTHOR CONTRIBUTIONS

The author J.S. was involved in the conception and design of the study, analyzing the data, and preparing the first draft of the manuscript. The author revised the draft manuscript and approved the final concept. The author agrees to be accountable for all aspects of the work in ensuring questions relating to the accuracy and integrity of any part of the work are appropriately investigated and resolved.

## FUNDING INFORMATION

This work was supported by JSPS KAKENHI Grant Number 23K10579.

## CONFLICT OF INTEREST STATEMENT

None declared.

## ETHICS STATEMENT

All procedures were approved by the Animal Care and Use Committee of Hokkaido University of Education (No. 1, approved on 2024/4/3).

## Supporting information


Figures S1–S12.

## Data Availability

Data file that supports the present results are available at https://docs.google.com/spreadsheets/d/1MhqFx0BP5Sh52s3iZFGnGLql9fpetcbx/edit?usp=sharing&ouid=103780764902759483879&rtpof=true&sd=true.
