# Supplementary material for: Exercise performance in well‐trained male mice is promoted by intermittent hyperoxia via improving metabolic properties and capillary profiles
Source: Physiol Rep. 2025 Apr 22;13(8):e70341. doi: 10.14814/phy2.70341 (PMC12012744; doi:10.14814/phy2.70341)

Figure S1-1

Molecular weight marker: Precision Plus Protein™ All Blue Prestained Protein Standards #1610373

Complete blot images shown in Figure 14

The lanes used in the figure

SOD1 (Figure 14A)

Total protein images after electrophoresis followed by trans-blot to membrane used as loading control

Specific bands of target protein detected after immunostaining

SOL

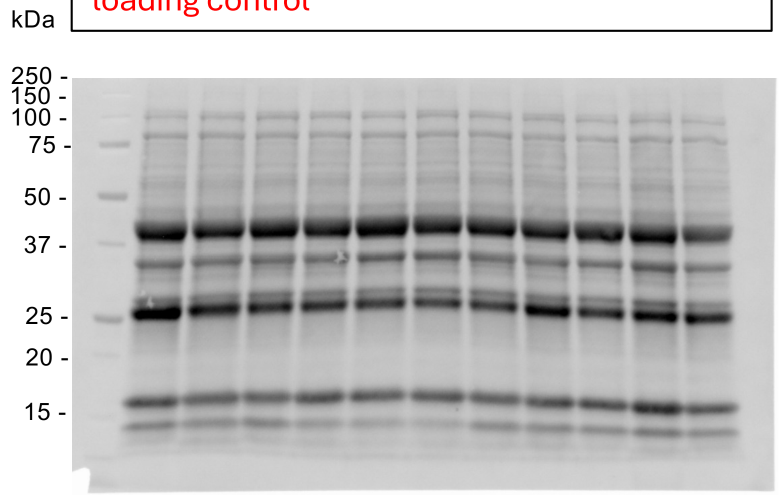

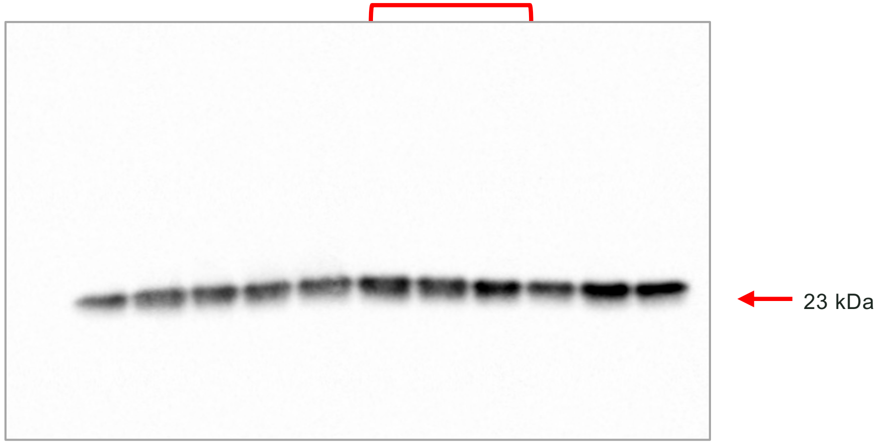

Gr

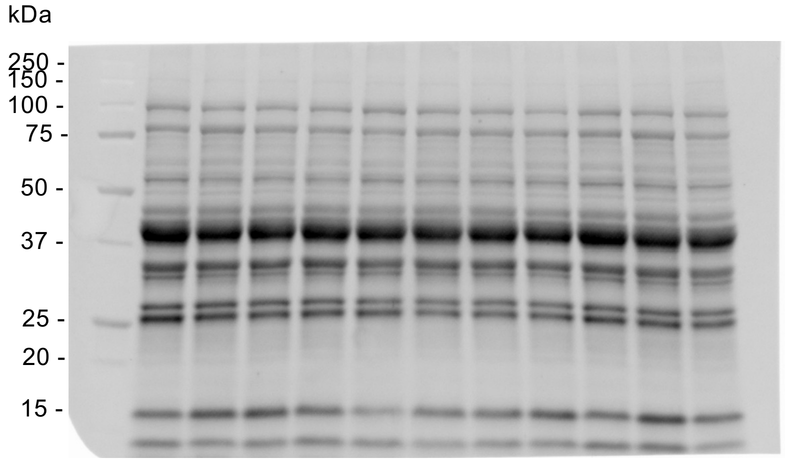

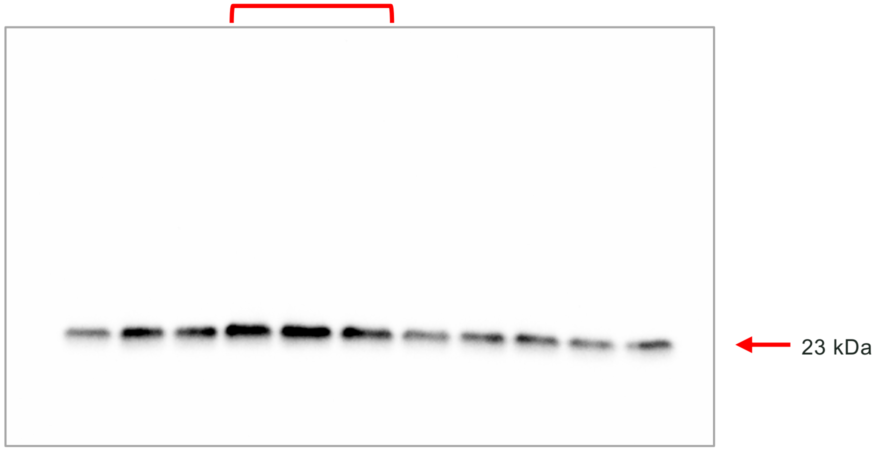

Figure S1-2

Molecular weight marker: Precision Plus Protein™ All Blue Prestained Protein Standards #1610373

Complete blot images shown in Figure 14

The lanes used in the figure

SOD1 (Figure 14A)

Total protein images after electrophoresis followed by trans-blot to membrane used as loading control

Specific bands of target protein detected after immunostaining

Gw

kDa
250 -
150 -
100 -
75 -
50 -
37 -
25 -
20 -
15 -

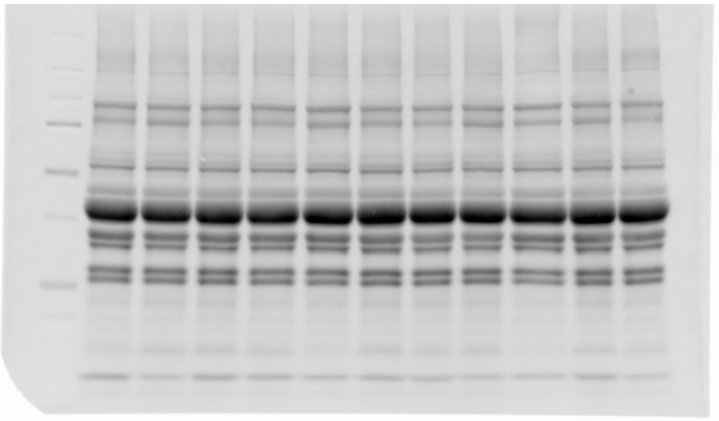

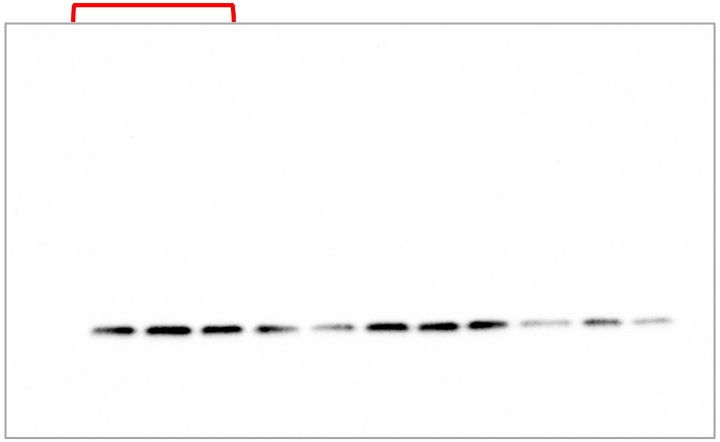

← 23 kDa

PL

kDa
250 -
150 -
100 -
75 -
50 -
37 -
25 -
20 -
15 -

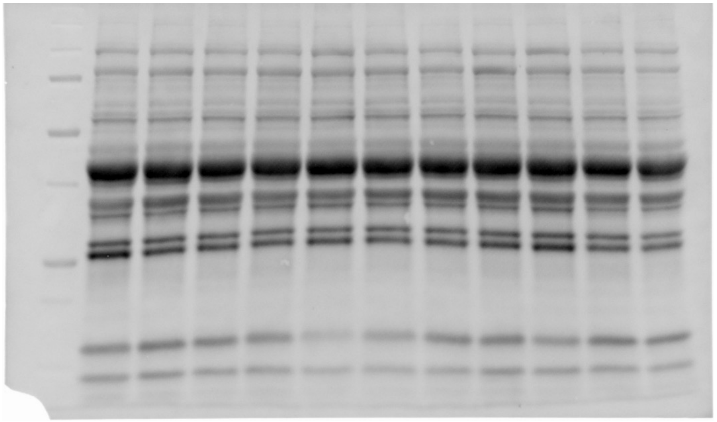

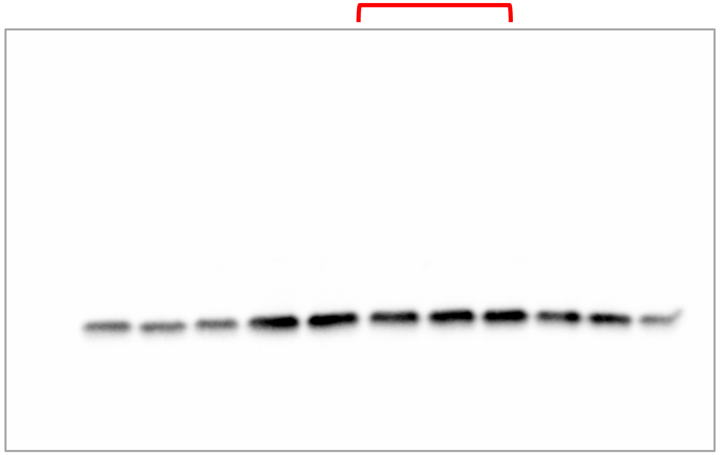

← 23 kDa

Figure S1-3

Complete blot images shown in Figure 14

The lanes used in the figure

Molecular weight marker: Precision Plus Protein™ All Blue Prestained Protein Standards #1610373

SOD1 (Figure 14A)

Total protein images after electrophoresis followed by trans-blot to membrane used as loading control

Specific bands of target protein detected after immunostaining

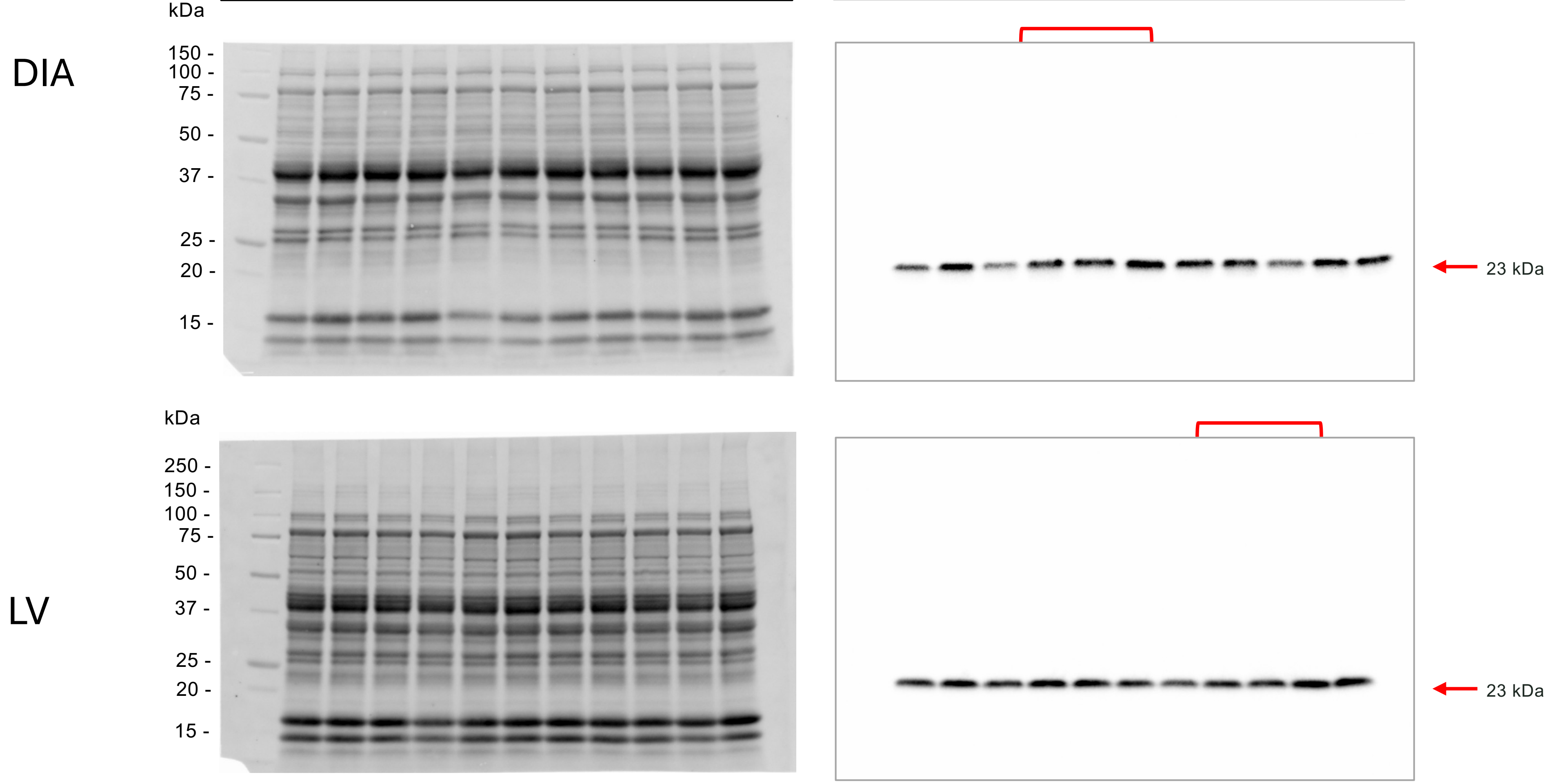

Figure S1-4

Complete blot images shown in Figure 14

The lanes used in the figure

Molecular weight marker: Precision Plus Protein™ All Blue Prestained Protein Standards #1610373

CAT (Figure 14B)

Total protein images after electrophoresis followed by trans-blot to membrane used as loading control

Specific bands of target protein detected after immunostaining

SOL

kDa

250 -
150 -
100 -
75 -
50 -
37 -
25 -
20 -
15 -

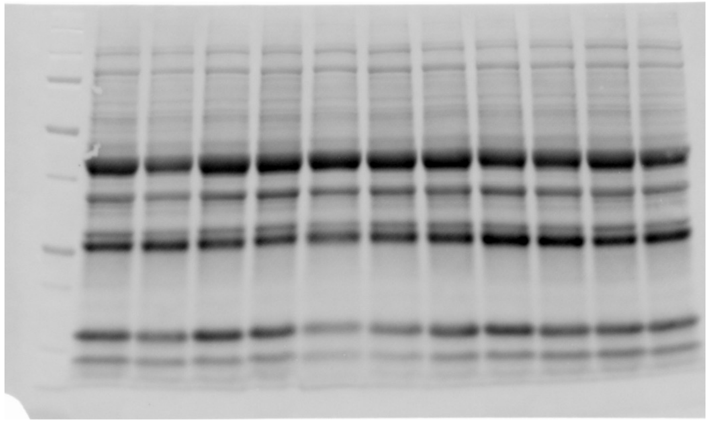

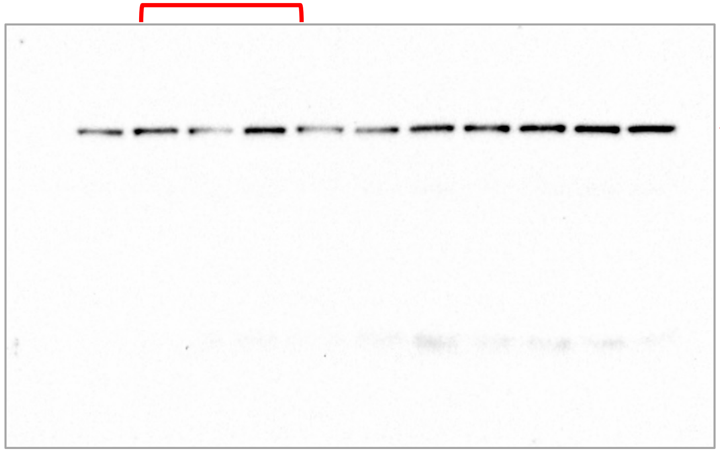

64 kDa

Gr

kDa

250 -
150 -
100 -
75 -
50 -
37 -
25 -
20 -
15 -

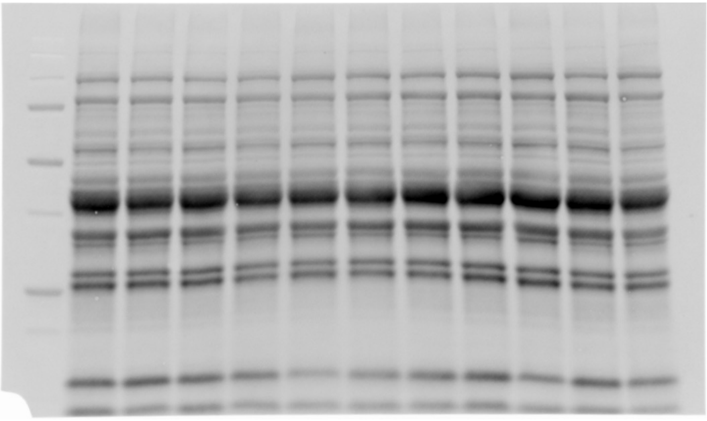

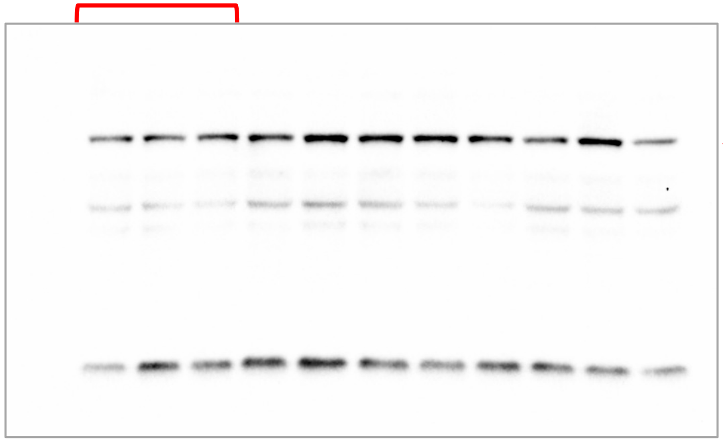

64 kDa

Figure S1-5

Molecular weight marker: Precision Plus Protein™ All Blue Prestained Protein Standards #1610373

Complete blot images shown in Figure 14

The lanes used in the figure

CAT (Figure 14B)

Total protein images after electrophoresis followed by trans-blot to membrane used as loading control

Specific bands of target protein detected after immunostaining

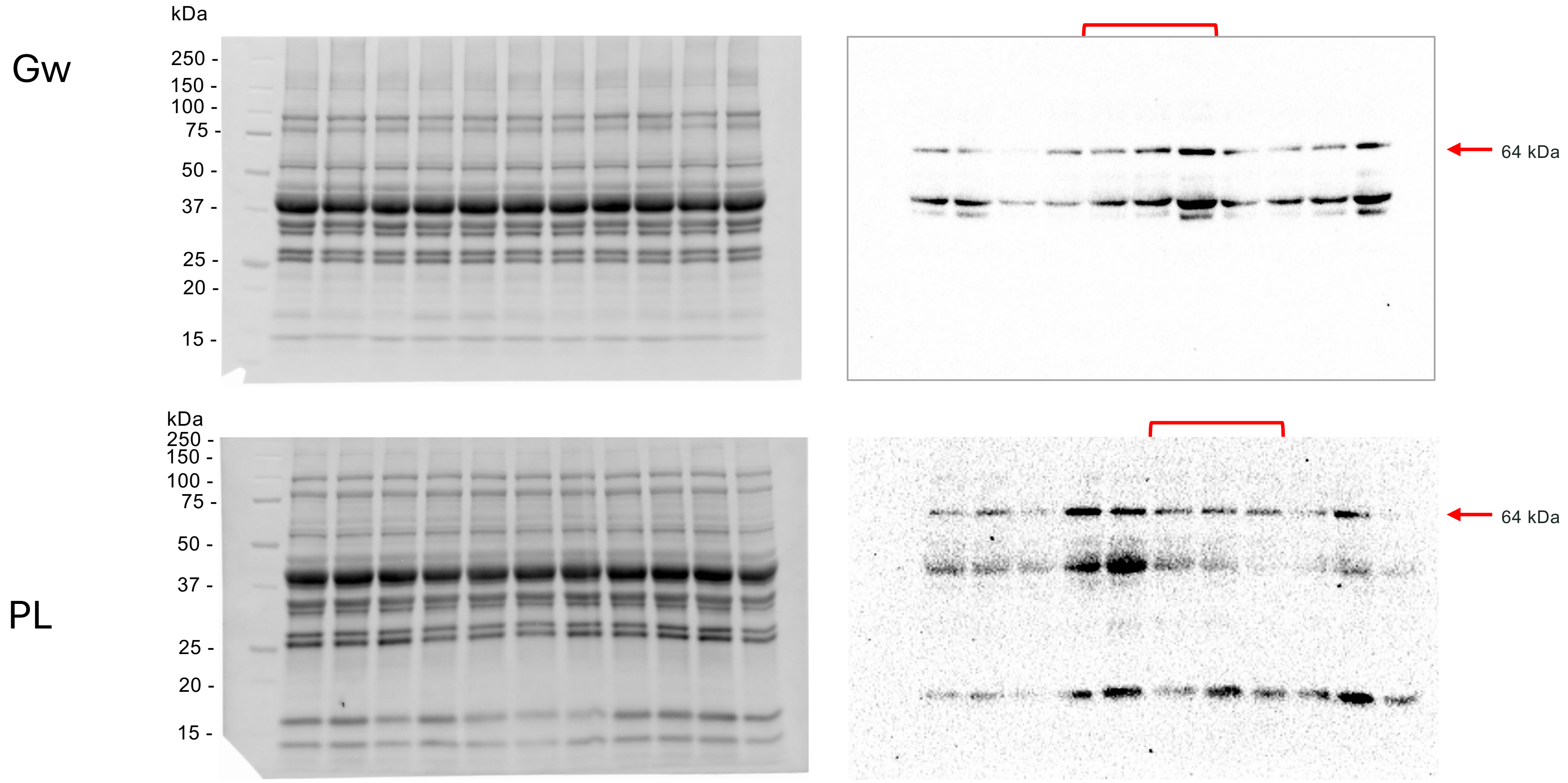

Figure S1-6

Complete blot images shown in Figure 14

The lanes used in the figure

Molecular weight marker: Precision Plus Protein™ All Blue Prestained Protein Standards #1610373

CAT (Figure 14B)

Total protein images after electrophoresis followed by trans-blot to membrane used as loading control

Specific bands of target protein detected after immunostaining

DIA

kDa

250 -
150 -
100 -
75 -
50 -
37 -
25 -
20 -
15 -

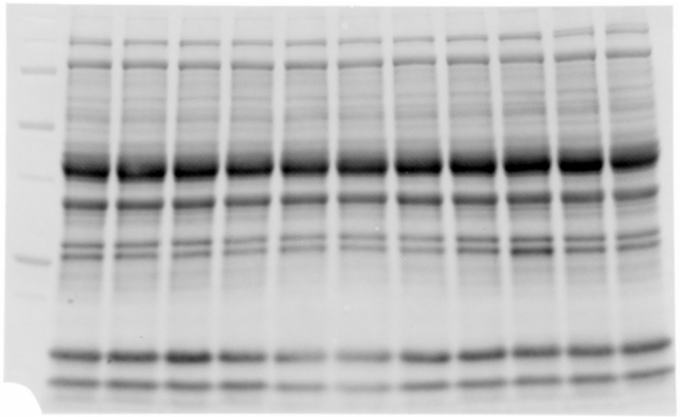

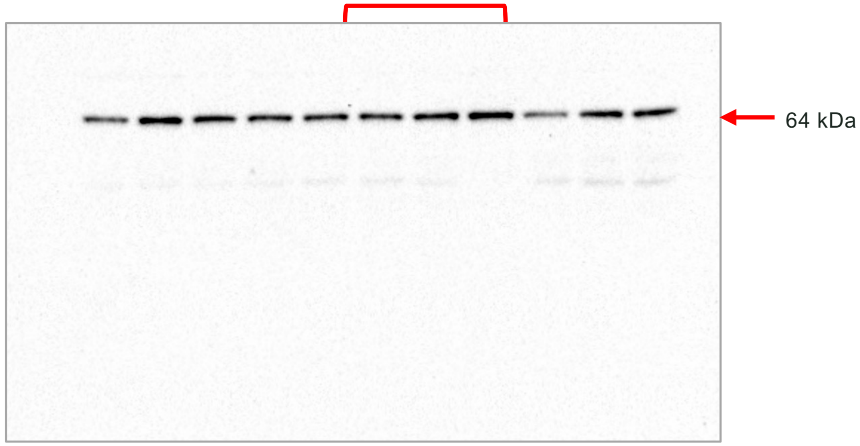

64 kDa

LV

kDa

250 -
150 -
100 -
75 -
50 -
37 -
25 -
20 -
15 -

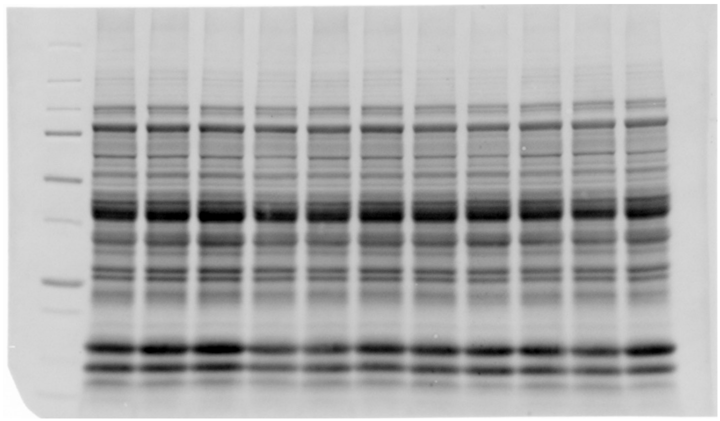

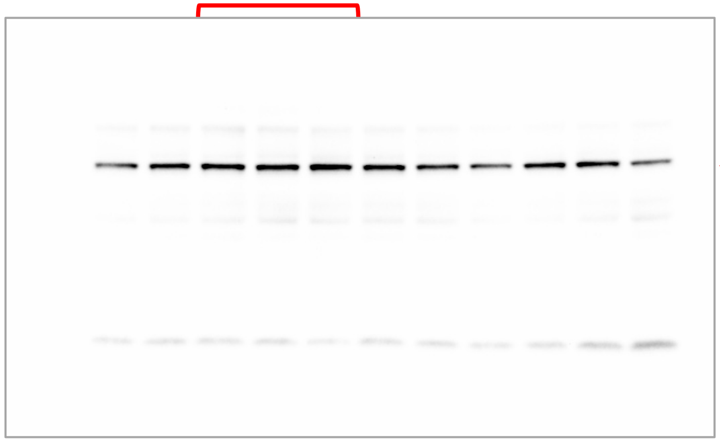

64 kDa

Figure S1-7

Complete blot images shown in Figure 14

The lanes used in the figure

GPX1 (Figure 14C)

Molecular weight marker: Precision Plus Protein™ All Blue Prestained Protein Standards #1610373

Total protein images after electrophoresis followed by trans-blot to membrane used as loading control

Specific bands of target protein detected after immunostaining

SOL

kDa

250 -
150 -
100 -
75 -
50 -
37 -
25 -
20 -
15 -

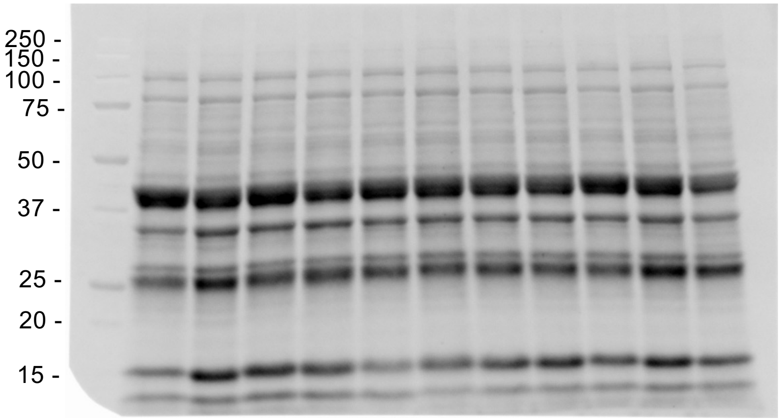

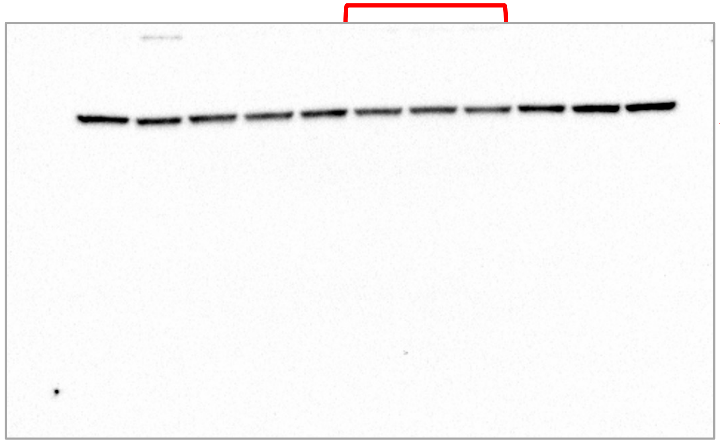

83 kDa

Gr

kDa

250 -
150 -
100 -
75 -
50 -
37 -
25 -
20 -
15 -

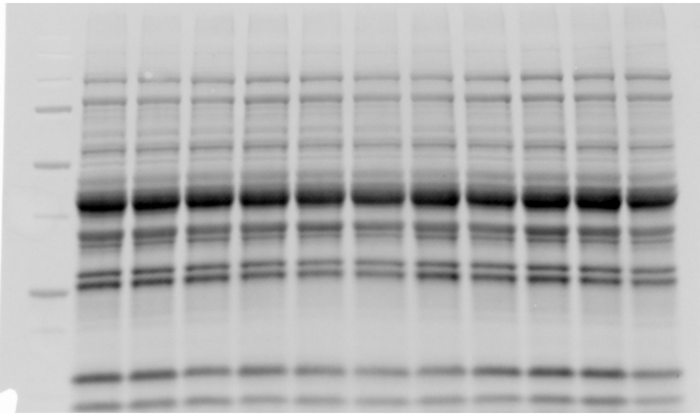

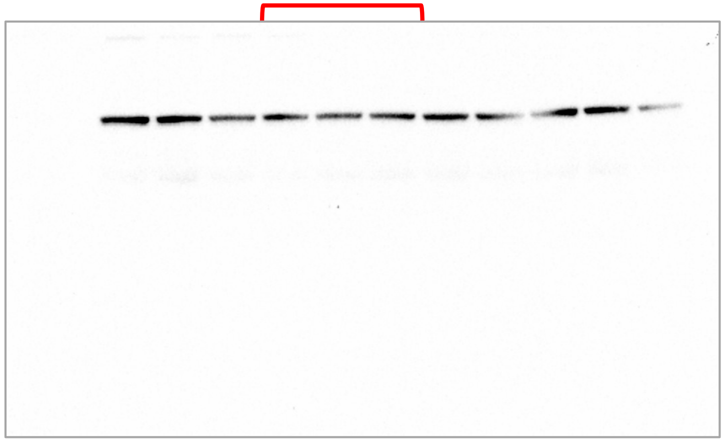

83 kDa

Figure S1-8

Complete blot images shown in Figure 14

The lanes used in the figure

Molecular weight marker: Precision Plus Protein™ All Blue Prestained Protein Standards #1610373

GPX1 (Figure 14C)

Total protein images after electrophoresis followed by trans-blot to membrane used as loading control

Specific bands of target protein detected after immunostaining

Gw

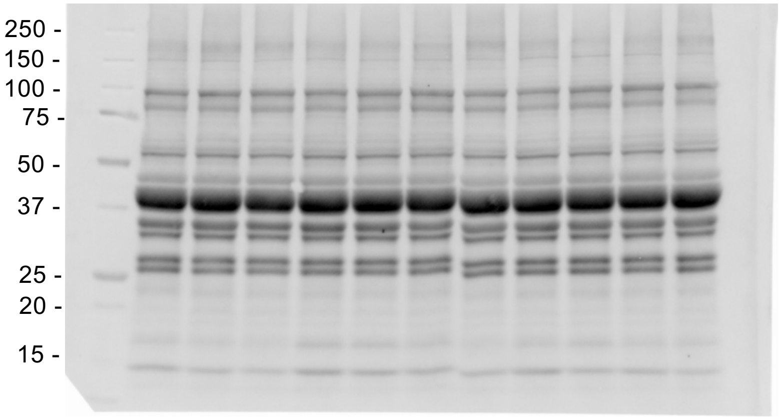

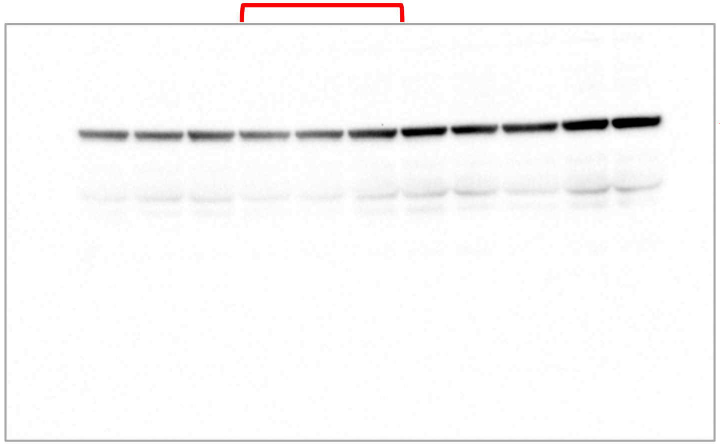

PL

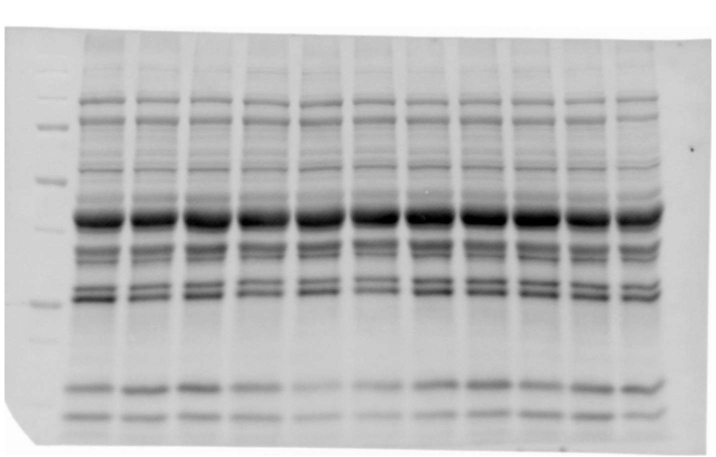

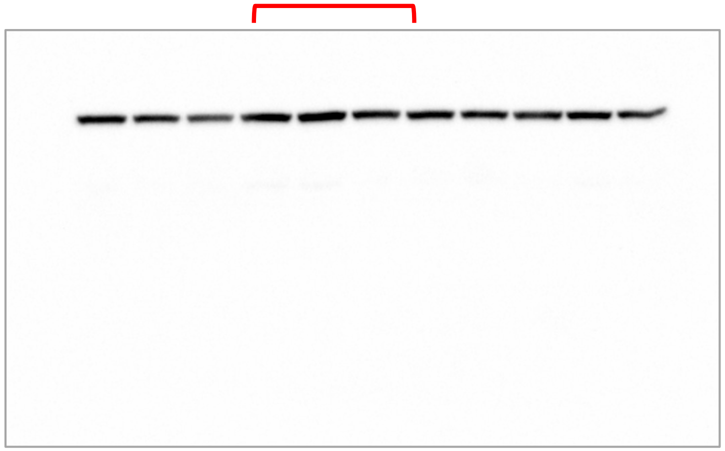

Figure S1-9

Complete blot images shown in Figure 14

The lanes used in the figure

Molecular weight marker: Precision Plus Protein™ All Blue Prestained Protein Standards #1610373

GPX1 (Figure 14C)

Total protein images after electrophoresis followed by trans-blot to membrane used as loading control

Specific bands of target protein detected after immunostaining

DIA

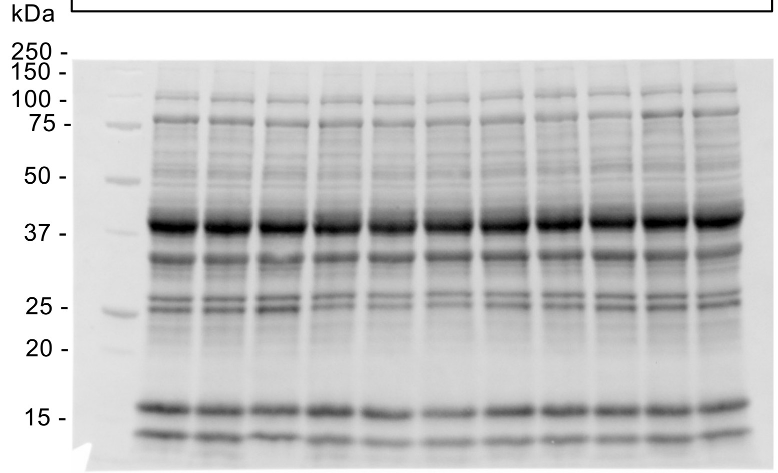

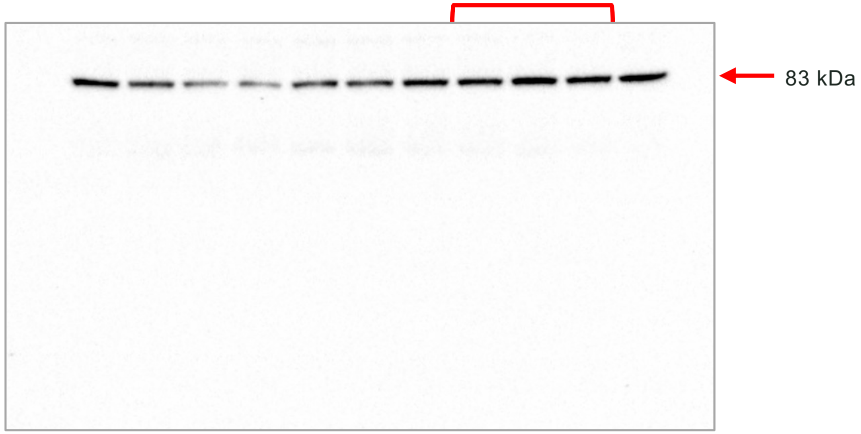

LV

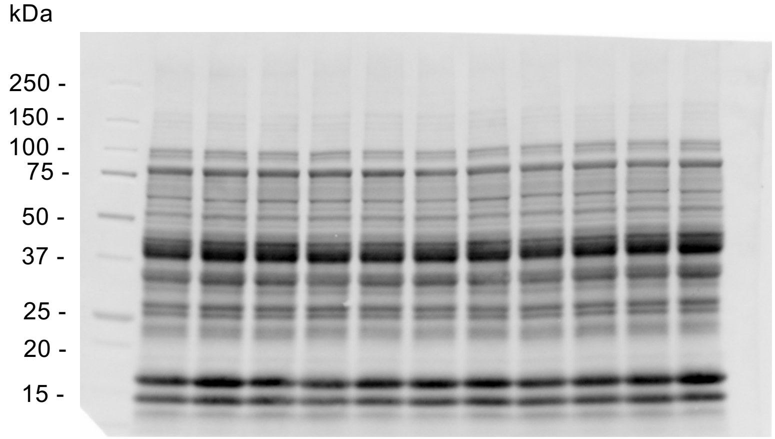

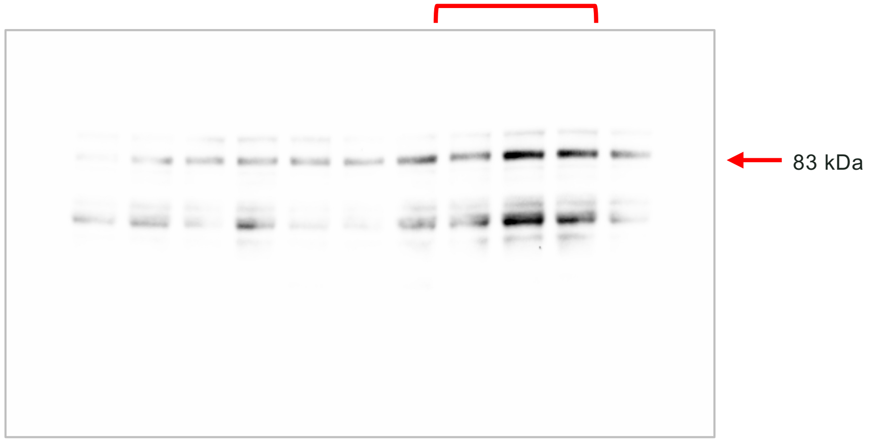

Figure S1-10 Previously published image by the author

Suzuki J. Endurance exercise under short-duration intermittent hypoxia promotes endurance performance via improving muscle metabolic properties in mice. Physiol Rep 10: 1–19, 2022. doi: 10.14814/phy2.15534.

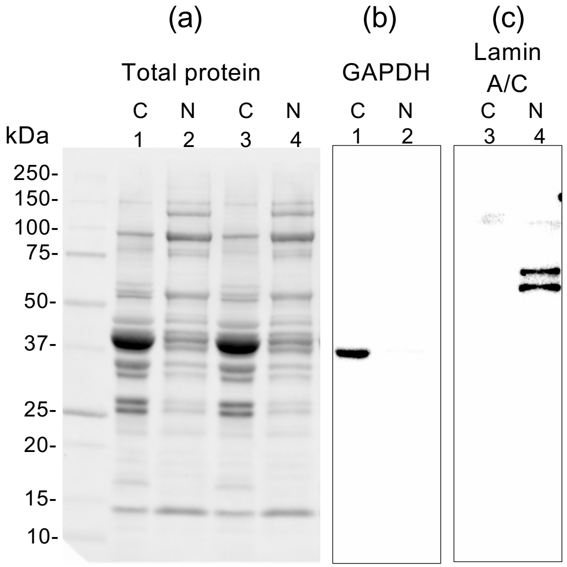

Figure S2
The efficacy of cytoplasmic or nuclear protein separation was confirmed by Western blot. (a) total protein after trans-blot to a membrane, (b) immunoblot image using anti-GAPDH antibody (cytoplasmic marker), (c) immunoblot image using anti-Lamin A/C antibody (nuclear marker). C and N, cytoplasmic and nuclear proteins, respectively.

Figure S1-11

Molecular weight marker: Precision Plus Protein™ All Blue Prestained Protein Standards #1610373

Complete blot images shown in Figure 15

Nuclear NT-PGC1α (Figure 15)

The lanes used in the figure

Total protein images after electrophoresis followed by trans-blot to membrane used as loading control

Specific bands of target protein detected after immunostaining

SOL

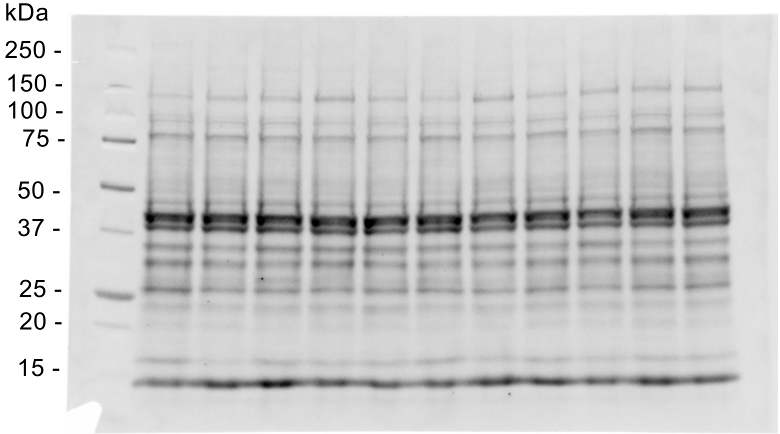

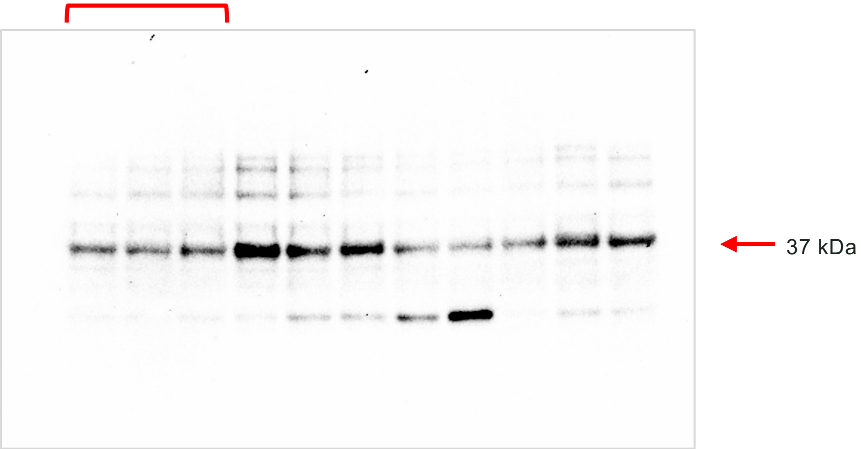

Gr

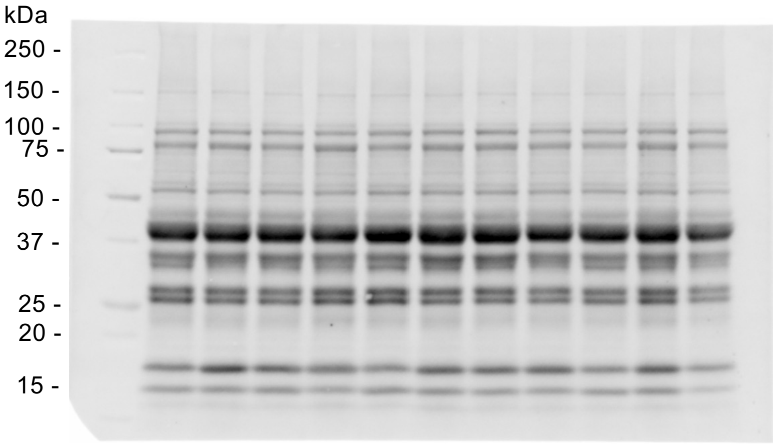

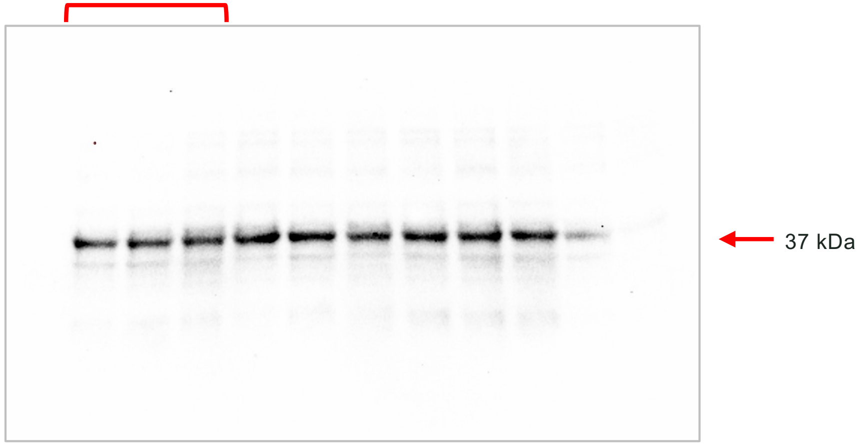

Figure S1-12

Complete blot images shown in Figure 15

Nuclear NT-PGC1α (Figure 15)

The lanes used in the figure

Molecular weight marker: Precision Plus Protein™ All Blue Prestained Protein Standards #1610373

Total protein images after electrophoresis followed by trans-blot to membrane used as loading control

Specific bands of target protein detected after immunostaining

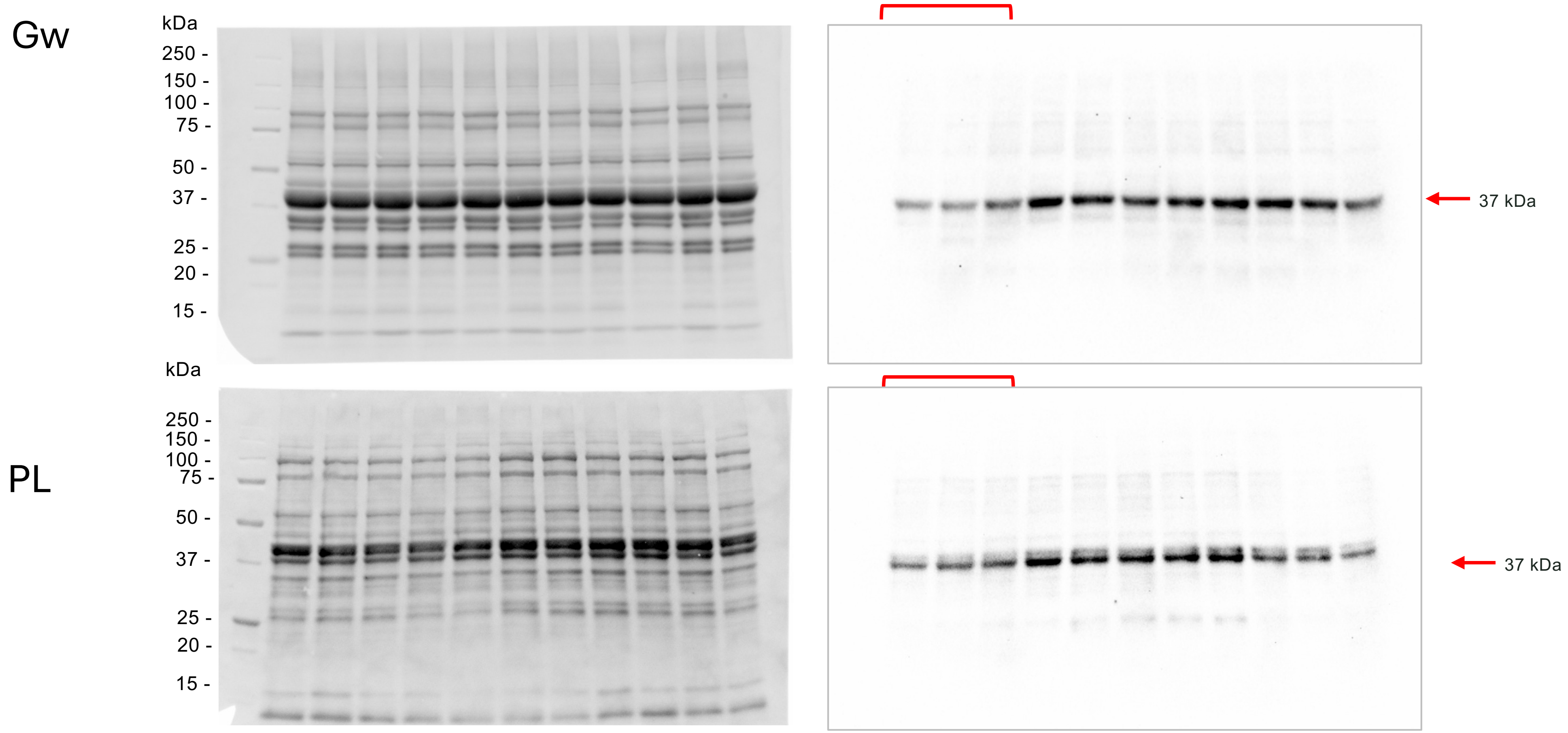

Supplement: Supplementary file 1 — Figures S1–S12. [file PHY2-13-e70341-s001.pdf]
